# AI-based planning for DIEAP flap procedures: exploring foundation models for artery perforators analysis

**DOI:** 10.3389/fmed.2026.1757637

**Published:** 2026-03-13

**Authors:** Matilde Andrade, Nuno Loução, David Pinto, Tiago Marques, Ricardo Vigário, Pedro Gouveia, João Santinha

**Affiliations:** 1 Digital Surgery Lab, Breast Cancer Research Program, Champalimaud Foundation, Lisbon, Portugal; 2 Department of Physics, NOVA School of Science and Technology, Lisbon, Portugal; 3 Breast Unit, Champalimaud Clinical Centre, Champalimaud Foundation, Lisbon, Portugal; 4 Faculty of Medicine, University of Lisbon, Lisbon, Portugal

**Keywords:** computer vision, deep learning, DIEAP flap, foundation models, preoperative planning, vessel segmentation, medical imaging

## Abstract

**Introduction:**

Breast reconstruction using the Deep Inferior Epigastric Artery Perforator (DIEAP) flap is the gold standard for autologous procedures, but its success relies on challenging preoperative planning. Identifying perforator vessels from Computed Tomography Angiography (CTA) images is currently a manual, labor-intensive, and variable process. This study's objective was to assess, fine-tune and validate an automated, end-to-end model-driven pipeline for the segmentation and quantitative analysis of perforator vessels to enhance planning efficiency and consistency.

**Methods:**

We developed a novel pipeline that first uses computer vision algorithms to extract anatomical priors and generate initial vessel centerlines from CTA data. These centerlines were then used as spatial prompts to guide a Deep Learning (DL) segmentation model. We benchmarked three state-of-the-art foundation models (SAM 2, MedSAM-2, and nnInteractive) in a zero-shot setting. The best-performing model, nnInteractive, was subsequently fine-tuned on our clinical dataset using a connectivity-aware compound loss incorporating Skeleton Recall Loss (SRL) to preserve vessel topology.

**Results:**

The fine-tuned nnInteractive model demonstrated significantly improved performance on a held-out test set of nine patients, increasing the mean Dice Similarity Coefficient (DSC) from a 0.174 zero-shot baseline to 0.265. Qualitatively, the fine-tuned model produced more anatomically plausible and continuous vessel segmentations compared to the baseline. Furthermore, the automated pipeline successfully quantified critical surgical planning metrics from the segmentations, including the perforators' intramuscular path length and their distance to the umbilicus.

**Conclusion:**

This study demonstrates the feasibility of an end-to-end, artificial intelligence (AI)-driven workflow for perforator mapping in DIEAP flap planning. The use of foundation models guided by anatomical priors and enhanced with topology-aware fine-tuning establishes a robust method for reducing manual annotation burden and improving consistency. This automated pipeline is a promising tool to support more efficient and reliable preoperative planning, ultimately poised to improve surgical outcomes.

## 1 Introduction

Breast cancer (BC) affects approximately 2.3 million people annually and remains the most commonly diagnosed cancer in women (1). In about 30% of cases, breast cancer treatment involves a mastectomy, where the entire breast is removed. Additionally, many women diagnosed with the BRCA genetic mutation, which significantly increases the risk of breast cancer, opt for risk-reducing mastectomy (2). While some patients might choose this procedure due to the fear of recurrence (3), Breast Conserving Surgery (BCS) remains a common choice for patients who prefer to maintain their body image (4, 5). When mastectomy is performed, many patients seek Breast Reconstruction (BR) intended to restore breast form, aiming for a natural and durable mound that can mature and change with the patient over time (6).

Breast reconstruction can be performed immediately, in the same surgery as the mastectomy, or there can be a delayed reconstruction done months or years after the first surgery (7), achieved via prosthetic breast implants or autologous tissue flaps, for which the Deep Inferior Epigastric Artery Perforator (DIEAP) flap procedure is the gold standard practice (8). This procedure involves the harvesting of excess skin and subcutaneous fat from the lower abdomen, the flap, while carefully dissecting perforator arteries and preserving the abdominal muscle and its nerves. Microvascular anastomosis is then performed between the deep inferior epigastric vessels and the internal mammary or the thoracodorsal vessels, at the time of the flap transfer (2), to ensure adequate blood supply to the transferred tissue.

Despite the popularity of implant-based BR, it is not always a feasible option (2). In cases where post-mastectomy radiation therapy is required, implant-based reconstruction carries higher risks of poor healing and complications, making autologous tissue reconstruction a preferable alternative. The DIEAP flap procedure offers several advantages, including natural-looking, long-lasting results, a reduced risk of rejection, and a lower overall complication rate. However, drawbacks such as lengthy preoperative planning, extended surgical time, and longer recovery periods pose significant challenges as the procedure demands a high level of surgical expertise (2, 9–11). A critical and time-consuming component of this procedure lies in identifying and selecting the optimal perforator vessels, which are crucial for ensuring flap viability.

Preoperative planning for DIEAP flap reconstruction relies heavily on Computed Tomography Angiography (CTA), which has become the gold standard for perforator mapping. CTA provides a comprehensive anatomical description of the perforator origin, intramuscular and subcutaneous course, fascial penetration, and skin emergence. The process involves intravenous contrast injection followed by high-resolution CT scanning, resulting in detailed three-dimensional visualization of the vascular anatomy. Careful optimization of contrast timing and injection protocols is essential to enhance the visibility of perforators while preserving the clarity of adjacent structures (12–14).

Following image acquisition, surgeons and radiologists typically analyze CTA datasets using Maximum Intensity Projection (MIP) tools within the hospital's Picture Archiving and Communication System (PACS). During this manual review, each perforator is annotated and characterized in terms of caliber, fascial intersection point, and distance from the umbilicus. Supplementary Figure S1 illustrates the process for these measurements. This evaluation, which can take up to 2 h per patient, is repeated for multiple perforators, commonly ranging from six to ten per case. Perforators are then assessed based on clinical criteria such as diameter (ideally ≥1.5*mm*), centrality (proximity to the periumbilical region), and intramuscular course length (preferably < 4*cm*). However, this manual process is both labor-intensive and operator-dependent, often subject to variability in interpretation and measurement, and prone to inconsistencies between preoperative imaging and intraoperative findings. In this case, tools to enhance the identification and characterization of perforators could serve the purpose of facilitating this preoperative planning, maximizing efficiency, and minimizing discrepancy. By providing surgeons with detailed and reliable maps of the perforator vessels, automated segmentation could not only alleviate the burden on radiological teams but also reduce variability and improve the consistency between preoperative and intraoperative findings, ensuring more reliable identification of the most suitable perforators, thereby enhancing surgical outcomes.

Previous research has explored different computational methods to automate vessel segmentation in CTA, moving away from labor-intensive manual drafting. Early approaches primarily utilized traditional image processing techniques, such as region-growing algorithms, to enhance and extract vascular structures (15). In the specific context of DIEAP planning, studies have employed thresholding and connectivity analysis to visualize perforators (13). More recently, Deep Learning (DL) architectures, particularly Convolutional Neural Networks (CNNs), have demonstrated superior performance in segmenting complex vascular networks by learning hierarchical features directly from the data (16–18). However, applying these models to perforator mapping remains challenging due to the small caliber of the vessels, the high variability in abdominal vascular anatomy, and the intensive requirement for large, expertly annotated datasets. While recent iterations have integrated attention mechanisms to focus on fine structures (19, 20), there remains a distinct lack of generalizable, “foundation-style” models capable of providing the robust, end-to-end characterization of the perforators required for surgical decision-making.

In this study, we assess prompt-based foundation models associated with computer vision methods to automate the extraction of metrics used for DIEAP flap procedures' planning. To achieve this in the next section we will show the process used to develop a robust, automated foundation model (FM)-based perforator segmentation pipeline that can evolve and improve over time through refinement and continual learning techniques. Additionally, we also describe the automated computational framework to accurately determine the intramuscular path length of perforators, while automating existing methods for computing distance to the umbilicus of the perforators' fascial intersection points. These will help to significantly reduce the time required for preoperative planning in DIEAP flap procedures.

## 2 Materials and methods

### 2.1 Dataset

This study was conducted using a curated dataset of 30 high-resolution abdominal CTA scans obtained from patients with breast cancer, imaged at the Breast Unit of the Champalimaud Clinical Centre. Scans met inclusion requirements of adequate quality, complete imaging sequences, and availability of the required MonoE40/45keV reconstructions. Cases with artifacts, incomplete studies, or lacking the required reconstructions were excluded. Of the 30 scans, 20 were used for zero-shot evaluation and fine-tuning, and 10 were reserved exclusively for testing. All patient data was handled in strict compliance with data protection and privacy protocols of the Champalimaud Foundation. Prior to analysis, imaging data underwent a rigorous and automated deidentification process to ensure the removal of Personally Identifiable Information (PII). The image data pipeline supporting this study was built upon a robust, profile-based anonymization architecture developed to facilitate the safe collection, processing, and dissemination of clinical imaging data for research purposes (21). Upon ingestion, the DICOM Gateway Anonymizer (DGA) component automatically applies a configurable anonymization profile that strips identifying information from DICOM metadata and pixel content and forwards the anonymized scans to a separate, research-dedicated PACS, isolated from the clinical environment. After deidentification, the volumetric scans were converted into the NRRD format to facilitate research and processing with the computational tools employed.

The manual annotations were designed to serve as reference standards for validating the final outputs of the vascular mapping pipeline. To ensure consistency and clarity in labeling, each annotated volume was systematically divided into three separate segmentations, each capturing distinct anatomical elements relevant to the vascular mapping pipeline. Full annotation protocol, including label conventions, imaging series used, file formats, folder structure, quality-control procedures, and examples of annotated volumes, is provided in Supplementary Section 2. The dataset originating from these annotations provides a high-quality, ethically sourced, and expertly annotated resource for the exploration and validation of methods targeting the robust mapping and segmentation of vascular structures. It serves as the empirical foundation for the approaches described in subsequent sections of this work.

### 2.2 Overall pipeline

**Figure 1:**
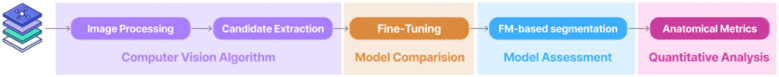
Overall pipeline representation.

The overall pipeline (Figure 1) is organized into four main components, each contributing to the accurate identification and characterization of perforator vessels from the CTA scans. It begins with a computer vision module that applies classical image processing to enhance vascular structures, segment relevant abdominal tissues, and extract initial perforator candidates with their centerlines, which serve as anatomical priors. These centerlines are then used in the model comparison stage, where three state-of-the-art foundation models are benchmarked in a zero-shot setting to identify the most suitable one for refinement. The selected model is subsequently fine-tuned and evaluated in the model assessment stage, allowing it to leverage both the raw images and extracted centerlines to produce more complete and anatomically consistent segmentations. Finally, a quantitative analysis stage uses the refined, fine-tuned, results to compute clinically meaningful measurements, including intramuscular path length and the distance from the perforator's fascial emergence to the umbilicus.

#### 2.2.1 Computer vision algorithm

This stage provides the initial anatomical priors used to support and constrain the later foundation-model–based segmentation. It focuses on narrowing down the regions of interest by enhancing vascular visibility, isolating relevant abdominal tissues, and extracting preliminary perforator candidates.

**Figure 2:**
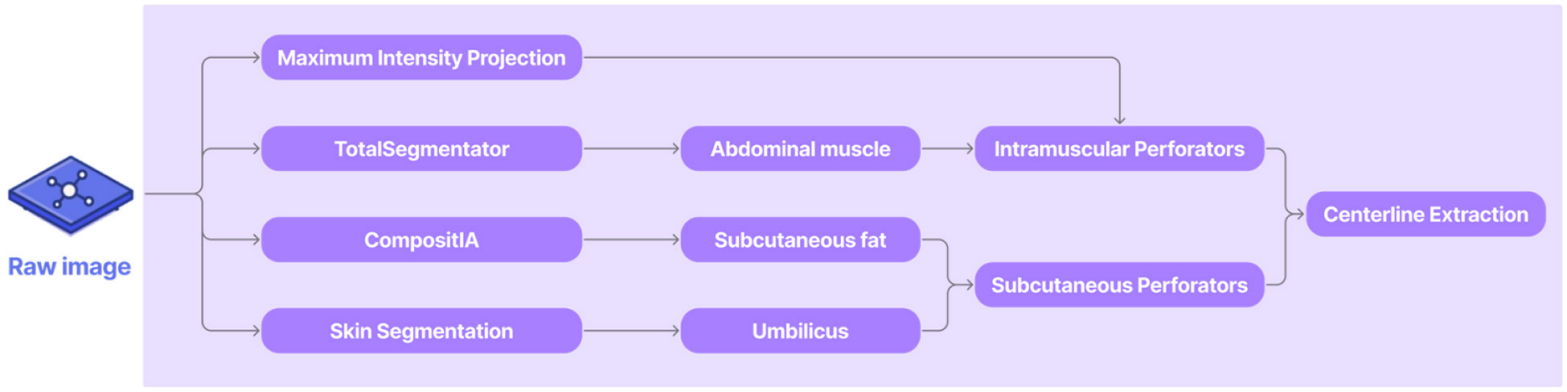
Overview of the computer vision (CV) algorithm.

As illustrated in Figure 2, the pipeline integrates several classical and deep-learning imaging components, including maximum intensity projection (MIP), skin segmentation and umbilicus detection, and soft-tissue segmentation using the CompositIA^1^ model (22). Together, these steps generate spatial cues that guide downstream refinement.

Originally, the *rectus abdominis* muscle was approximated using morphological operations on the CT volume. This was later replaced with a more robust approach based on TotalSegmentator (23), which directly segments the left and right rectus muscles in 3D. The fascia continued to be extracted via morphological computation, but now using the segmentation predictions of the updated model. The two main components of this stage are described below.

##### 2.2.1.1 Image processing

The image processing pipeline was designed to highlight vascular structures and generate anatomically meaningful masks for subsequent perforator estimation. CTA slices first underwent intensity-based filtering and enhancement to improve vessel contrast and boundary clarity. Tissue masks were then created to identify key anatomical structures, including:

- **Skin and umbilicus**, used as patient-specific geometric reference markers;
- **Abdominal wall and subcutaneous regions**, supporting the distinction between intramuscular and extramuscular perforators;
- ***Rectus abdominis* muscles**, robustly segmented using TotalSegmentator to improve consistency across scans.

These elements provide localized anatomical context, reduce the search space for potential perforators, and increase the reliability of the later deep-learning segmentation. A more detailed description of each component of the image processing workflow can be found in Supplementary Section 3.

##### 2.2.1.2 Centerline extraction

Following vessel enhancement and binary segmentation of candidate perforators, a centerline extraction procedure was applied to derive simplified, anatomically consistent paths representing the approximate 3D course of each vessel through the abdominal wall. These centerlines serve two main purposes: (i) they provide a simplified anatomical abstraction that facilitates clinical measurements and downstream geometric analysis, and (ii) they act as structural priors for the fine-tuned deep-learning model, helping to guide predictions along plausible vascular trajectories, particularly in challenging regions of low contrast or imaging noise. A detailed description of the centerline extraction algorithm is provided in Supplementary Section 3.7.

### 2.3 Model comparison (zero-shot benchmarking and fine-tuning)

**Figure 3:**
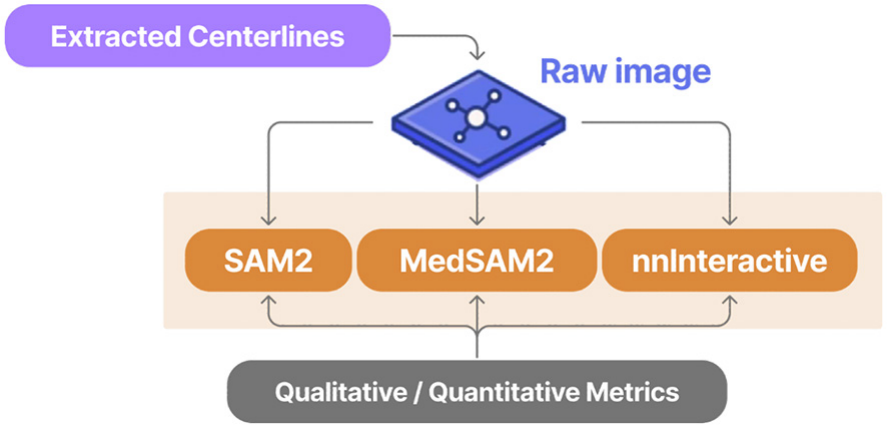
Overview of the model comparison stage where 3 foundation models are benchmarked in a zero-shot setting using centerline-guided prompting.

To assess the feasibility of using large foundation models for perforator segmentation, three state-of-the-art promptable models were evaluated in a zero-shot setting. Each model received the original CT images together with centerline-guided positive and negative prompts, allowing the quality of vessel localization and delineation to be examined. The overall benchmarking workflow is illustrated in Figure 3. Here, each model processes the same set of 20 CTA scans, using the previously extracted centerlines to generate slice-wise prompts. The outputs are then evaluated both qualitatively and quantitatively against the manually annotated ground truth segmentations to determine which model performs best in this specific application context. To formally assess whether the observed differences between models were statistically significant, a MANOVA was first conducted across all performance metrics. Subsequent one-way ANOVA tests were then performed for each individual metric, with *post-hoc* Tukey's pairwise comparisons applied to identify specific pairwise differences. This rigorous statistical approach ensures that the selection of the best-performing model is based on robust evidence rather than chance variations.

#### 2.3.1 Foundation model benchmarking and zero-shot evaluation

Performance was evaluated on 20 cases using both qualitative inspection and quantitative segmentation metrics. This comparison provided insight into how well each model aligned with vascular anatomy based solely on prompting, highlighting strengths and limitations in handling branching, vessel thinness, and contrast variability. From this evaluation, the most promising model was selected as the foundation for subsequent refinement, balancing overall segmentation performance with the ability to follow anatomically plausible vascular trajectories. Table 1 summarizes the key characteristics of each model, including backbone architecture, pretraining domain, dataset origin, prompting mechanisms, and whether domain-specific adaptations or medical fine-tuning were applied.

**Table 1:** Summary of foundation models evaluated in a zero-shot setting.

**Model name**	**Architecture**	**Domain**	**Dataset**	**Prompting strategy**	**Medical-specific tuning**
SAM 2 (34)	Hiera ViT (1024 × 1024) + FPN + MAM + mask decoder	Natural images, video	SA-1B, HQ-YouTube-VIS	Points, boxes, masks, free-form text	No
MedSAM-2 (35)	Hiera ViT (512 × 512) + FPN + MAM + simplified Mask decoder	Medical imaging (includes video)	Diverse 3D medical dataset (CT, MRI, PET, ultrasound, endoscopy)	Points, boxes	Yes
nnInteractive (36)	nnUNet + residual encoder	Medical imaging	Over 120 public 3D segmentation datasets (CT, MRI, PET, microscopy, and ultrasound)	Points, boxes, lasso, scribble	Yes

#### 2.3.2 Centerline-guided prompting

In this step, previously extracted vessel centerlines serve as the basis for defining 2D input prompts for the segmentation models, with each axial slice receiving a corresponding positive point located at the projected centerline location. This prompt is designed to anchor the model's attention to the likely position of the vessel within the image. As mentioned before, rather than directly selecting the centerline point as the prompt, a local intensity-based refinement mechanism is introduced. For every extracted coordinate, the algorithm searches for the highest intensity voxel within a small radius of 5 voxels, assumed to correspond to the lumen of the perforator vessel. This adjustment ensures better alignment of the prompt point with the vessel interior, accounting for potential small deviations in the centerline or partial volume effects. The goal is to guide the model with the prompts that are both geometrically accurate and radiologically consistent.

To further improve specificity and suppress false positives, negative prompts are also introduced. These are derived using geometric constraints, such as radial offsets from the centerline, and intensity-based rules, typically sampling regions near, but outside, the vessel boundary. This helps the model distinguish the true vessel from surrounding structures, especially in regions where contrast is weak or where vessel boundaries are ambiguous. Each slice is then processed independently using a promptable segmentation model, configured in single-mask mode to generate a binary prediction of the vessel region. These outputs are then stacked to reconstruct the full 3D segmentation of the vessel path.

**Figure 4:**
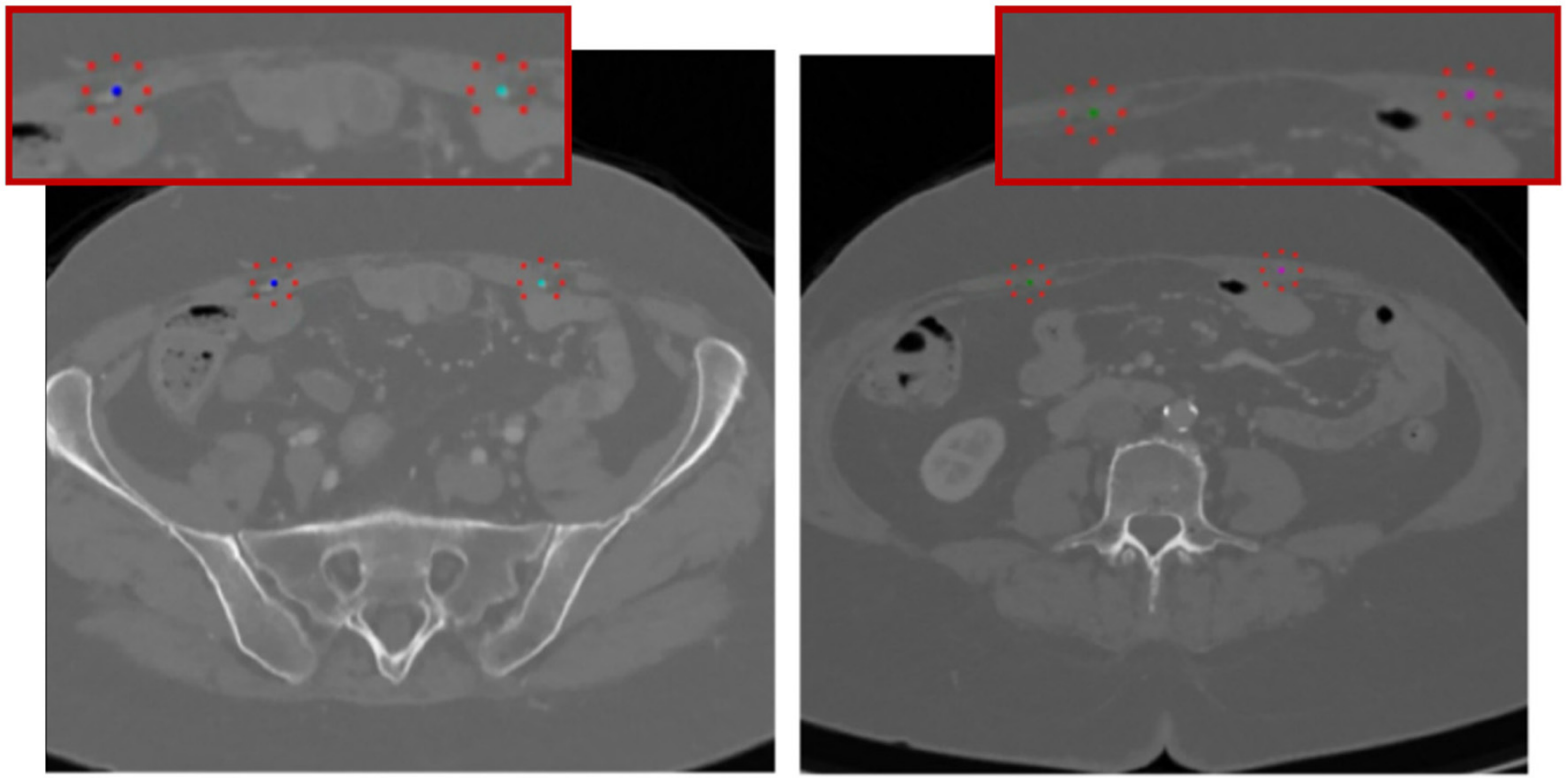
Representation of the centerline-guided prompting strategy, with ilustration of positive (dark and light blue, green, and pink) and negative inptus (red).

The prompting strategy, including both positive and negative inputs, is exemplified along two different anatomical regions in Figure 4.

### 2.4 Model assessment

**Figure 5:**
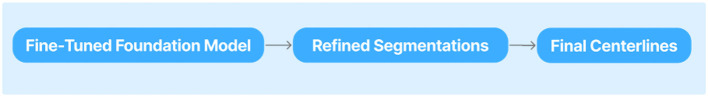
Representation of the model assessment stage where the best-performing model is fine-tuned using connectivity-aware loss functions and the same centerline-guided prompting to produce refined segmentations and the final centerlines for perforator anatomical quantitative analysis.

The chosen foundation model is fine-tuned using the annotated dataset and uses both the raw images and extracted centerlines during inference to improve anatomical accuracy and mitigate missing or fragmented vessel predictions. The overall model assessment and fine-tuning workflow, including the use of connectivity-aware loss functions and centerline-guided prompting to generate refined segmentations, is illustrated in Figure 5.

#### 2.4.1 Fine-tuning with connectivity-aware loss functions

Fine-tuning was performed on the best-performing model, with the subset of 20 cases previously used for zero-shot inference. Fine-tuning was conducted within the nnU-Net v2 framework using its default five-fold cross-validation strategy. This phase aimed to enhance the model's ability to accurately capture the intricate vessel structures by optimizing both volumetric accuracy and topological correctness. To achieve this, the SRL^2^ (24) was employed. The overall loss function started as a weighted combination of Dice Loss, CE Loss, and SRL, with weights set to [1, 1, 1]. These were later optimized with the PMOO (25, 26) to balance volumetric accuracy and topological correctness.

The fine-tuned model was then evaluated on a separate set of 10 previously unseen cases, which were not used during training or hyperparameter optimization, to assess performance improvements, using the same metrics applied during the initial comparison of the three models. This allowed for a direct quantification of the fine-tuning impact on segmentation quality and connectivity.

Table 2 summarizes the best-performing training hyperparameters, as determined by maximizing the mean validation Dice score. For a more detailed description of the optimization procedure and additional parameter tuning results, readers are referred to Supplementary Section 4.4.

**Table 2:** Summary of training hyperparameters.

**Patch size**	**Batch size**	**Initial weights**	**Epoch schedule**	**Patience**
[96, 192, 192]	4	Dice: 0.5, SRec: 0.33, CE: 0.17	Up to 150 epochs	20

#### 2.4.2 Validation and evaluation metrics

Accurate evaluation of segmentation models requires assessing both the volumetric overlap of predicted masks with ground truth and the precision of their boundaries. Overlap-based metrics capture how well the overall shapes and volumes match, while boundary-based metrics reveal discrepancies along the edges that can be critical in clinical contexts, especially when precise anatomical details are essential. Using both types of metrics provides a comprehensive understanding of model performance.

All model predictions, whether zero-shot or fine-tuned, were quantitatively compared against manually annotated ground truth segmentations using a comprehensive set of evaluation metrics. These included overlap-based metrics, Dice Similarity Coefficient (DSC) and Intersection over Union (IoU), boundary-based metrics, Hausdorff Distance (HD), and Average Surface Distance (ASD) (27), and one additional efficiency-based metric, Inference Time per Centerline (ITpC). In addition to these quantitative measures, a thorough visual inspection was conducted, focusing on edge and outlier cases, including missed detections, double-segmented branches, and irregular trajectories to provide qualitative validation.

### 2.5 Perforator quantitative analysis

**Figure 6:**
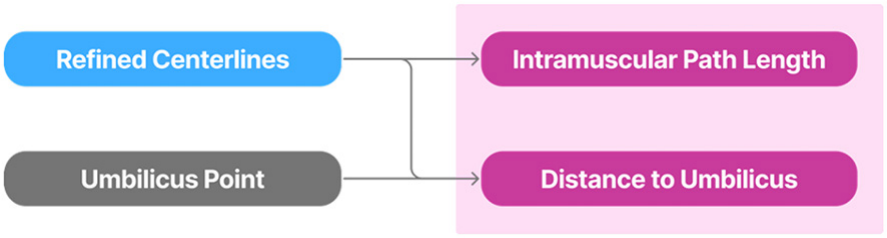
Representation of the quantitative analysis stage where clinically relevant anatomical metrics are computed from the predicted perforator segmentations.

The perforators obtained using the fine-tuned foundation model were then used to extract clinical relevant metrics guiding the planning of the DIEAP flap procedures, as illustrated in Figure 6. This process was fully automated and designed to translate anatomical segmentations into usable surgical insights, thereby enhancing the value of the algorithm for preoperative planning. Two primary anatomical descriptions are targeted: the length of the intramuscular path of the vessel, and the distance the fascial intersection point to the umbilicus, measured both horizontally and vertically.

Each of the following sections details the methodology used to compute these anatomical features, starting with centerline-based length estimation, followed by the distance to the umbilicus calculation. Finally, the specific challenges related to validating these measurements are discussed.

#### 2.5.1 Intramuscular path length

To estimate this metric in an automated and reproducible manner, a centerline-based analysis was implemented, allowing for robust measurement of the vessel's length while accounting for its 3D tortuosity. Following the completion of vessel segmentation, the refined centerline of each candidate perforator is extracted. However, for this computation, only the coordinates intersecting the muscle mask are used.

To compute the intramuscular path length, pairwise Euclidean distances between consecutive physical points along each extracted centerline are calculated and summed. This process yields the total 3D length of each perforator's intramuscular segment, expressed in millimeters, being fully automated and applied uniformly to all newly predicted masks, ensuring consistency and eliminating operator bias. This centerline-based approach to quantify perforator intramuscular length allows for high-resolution anatomical interpretation directly from segmentation masks, providing objective and scalable measurements suitable for surgical decision support.

#### 2.5.2 Distance to umbilicus

To compute distances of the perforators intersection with the fascia and the umbilicus in a fully automated and anatomically consistent manner, the centerline of each segmented perforator is extracted. The most superficial point along this centerline that lies within or near the fascia mask is identified as the fascial emergence point. This is defined as either a direct intersection with the fascia or the closest point within a 20-voxel proximity threshold along the Y-axis (anterior-posterior direction). This point serves as the representative fascial emergence coordinate for the perforator.

Once this reference point is established, its spatial relationship to the previously detected umbilicus landmark is quantified. The distance between this landmark and the perforator's fascial emergence point is decomposed into vertical and horizontal components. The vertical distance accounts for the difference in Z-axis (superior-inferior direction, i.e., axial slice index). The horizontal distance is computed in the axial plane based on the difference in X-axis (left-right) coordinates. The result is a pair of clinically interpretable distance values, vertical and horizontal, that precisely describe the location of each perforator relative to the umbilicus, aiding surgical mapping and preoperative planning. As with other anatomical features, this measurement pipeline is entirely automated and applied uniformly to all predicted segmentations.

#### 2.5.3 Validation

Validation of the computed anatomical features is critical to assess the clinical reliability of the proposed pipeline. However, unlike segmentation tasks, where ground truth annotations allow for standardized quantitative evaluation using metrics such as DSC and HD, the validation of the anatomical descriptors, such as intramuscular path length and distance to the umbilicus, presents distinct challenges due to the limited availability of direct quantitative references in clinical documentation.

To ensure internal consistency, the intramuscular path length metric is computed using the same centerline-based methodology described previously, applied not only to the predicted segmentation masks but also to the manually annotated ground truth. This parallel computation allows for the direct comparison of measurements derived from model outputs with those derived from expert segmentations. In the case of intramuscular path length, this strategy provides the only viable reference, as clinical reports rarely include exact numerical values for vessel lengths. Instead, these features are often described qualitatively using terms such as “long” or “short.” Consequently, validation of this result is inherently limited.

In contrast, the distance from the perforator's fascial intersection point to the umbilicus can be validated using radiological reports with manual measurements performed within the DICOM Viewers software.

To quantify agreement across all anatomical metrics, a set of descriptive and inferential statistics was employed. This includes the root mean square error (RMSE) and median absolute error (MdAE), accompanied by the mean absolute error (MAE) and interquartile range (IQR) to capture variability. This comprehensive validation approach aims not only to quantify accuracy but also to characterize the reliability of each anatomical descriptor within the context of preoperative planning.

## 3 Results

### 3.1 Zero-shot evaluation of segmentation models

The first stage of the evaluation focused on assessing the zero-shot performance of three foundation models (SAM 2, MedSAM-2, and nnInteractive) on the task of perforator vessel segmentation for 20 different CTA volumes. Each model was used directly without task-specific fine-tuning, enabling a fair comparison of their inherent generalization capabilities. Performance was quantified using volumetric overlap metrics, such as DSC and IoU, boundary accuracy metrics, like the ASD and HD, and a computational efficiency metric, ITpC. All models were evaluated on a NVIDIA RTX A6000 GPU with 48GB of VRAM. As perforators are thin tubular structures, minor voxel-level discrepancies can lead to substantial decreases in DSC/IoU scores, a well-documented issue when applying overlap-based metrics to small structures (27). Therefore, boundary-based metrics were also emphasized to provide a more nuanced understanding of segmentation quality. The results for each model are detailed below.

#### 3.1.1 SAM 2

On average, the SAM 2 model achieved a mean DSC of 0.095 ± 0.066, reflecting a very low degree of volumetric overlap between the predicted perforator segmentations and the manually annotated ground truth. The corresponding IoU was 0.051 ± 0.042, in line with expectations for such low DSC and vessel segmentation tasks, where the small caliber of the target structures and frequent branching increase the likelihood of partial mismatches.

Boundary-based metrics further contextualized segmentation accuracy. The mean and median ASD were 22.64 ± 10.55 mm and 20.25 mm, while the mean and median HD reached 95.38 ± 34.60 mm and 86.35 mm, highlighting the presence of large spatial deviations in the majority of cases. Notably, the wide range of values, from 53.58 mm to 215.32 mm, suggests that model performance was strongly case-dependent, with several outlier cases contributing to the overall error. The HD95 was 59.64 ± 22.29 mm and, although lower, still shows the underperformance of the model. ITpC yielded an average value of 53.73 ± 16.46 s. Supplementary Table S1 summarizes the results across all cases.

#### 3.1.2 MedSAM-2

The MedSAM-2 model achieved a mean DSC of 0.075 ± 0.094, with a corresponding IoU of 0.042 ± 0.056, as summarized in Supplementary Table S2. These results indicate a very low degree of segmentation overlap, comparable to SAM-2 model's reported performance for perforator vessel segmentation (Section 3.1.1).

Boundary-based assessments further illustrated the model's strengths and limitations. The mean ASD was 21.51 ± 10.04 mm, while the mean HD reached 86.16 ± 25.78 mm. Again, the full observed range 40.93–150.83 mm underscores the case-dependent variability. The HD95, which provides an estimate of boundary agreement, mitigating the effect of outliers, reached 60.93 ± 25.99 mm, still very high for the precision required for the procedure. ITpC reached an average of 154.67 ± 84.27 s, representing an increase of almost 188% compared to SAM 2.

#### 3.1.3 nnInteractive

This approach achieved a higher average DSC of 0.147 ± 0.095, with a corresponding IoU of 0.082 ± 0.057, suggesting improved volumetric overlap relative to SAM 2 and MedSAM-2. Detailed evaluation of the nnInteractive model is reported in Supplementary Table S3.

Surface distance metrics corroborated this trend. The mean ASD was reduced to 17.58 ± 8.95 mm, while the mean HD was 75.44 ± 25.87 mm, indicating closer boundary agreement compared to the previous models. The narrower observed range, 29.51–120.33 mm, further emphasize the model's more stable performance across cases. Importantly, the mean HD95 (50.14 ± 25.89 mm), with the median value of 44.54 mm, demonstrated a more robust boundary alignment with fewer extreme deviations, highlighting the effectiveness of nnInteractive in mitigating large outlier errors. ITpC averaged 99.6 ± 30.22 s, representing a considerable reduction compared to the prior MedSAM-2 model. Although slightly slower than SAM 2, this efficiency loss is compensated with the gain in accuracy.

#### 3.1.4 Comparative analysis of zero-shot performance

In terms of volumetric overlap, nnInteractive achieved the highest mean DSC (0.147 ± 0.095) and IoU (0.082 ± 0.057), outperforming both SAM 2 (DSC: 0.095 ± 0.066, IoU: 0.051 ± 0.042), and MedSAM-2 (DSC: 0.075 ± 0.094, IoU: 0.042 ± 0.056). Boundary accuracy further underscored the advantage of nnInteractive. The model achieved the lowest mean ASD (17.58 ± 8.95 mm) and HD (75.44 ± 25.87 mm), both improvements over MedSAM-2 (ASD: 21.51 ± 10.04 mm, HD: 86.16 ± 25.78 mm) and SAM 2 (ASD: 22.64 ± 10.55 mm, HD: 95.38 ± 34.60 mm). The HD95 confirmed this trend, with nnInteractive demonstrating substantially fewer extreme deviations than either alternative, reflecting superior boundary conformity. Computational demands varied markedly across models. MedSAM-2 incurred the longest inference times per centerline (mean: ~155 s, max: ~299 s), whereas SAM 2 was considerably faster (mean: ~54 s, max: ~95 s). nnInteractive fell between the two, with a mean ITpC of ~100 s and maximum of ~ 171 s.

**Figure 7:**
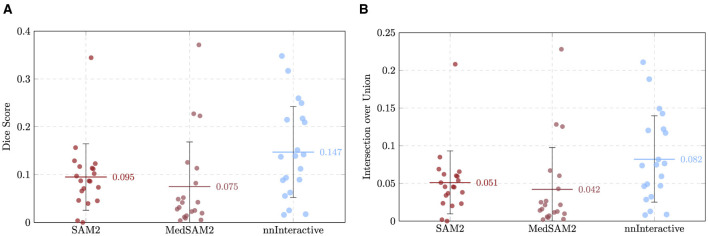
Comparison of the overlap metrics across the three models for each patient, with mean and standard deviation indicated. **(A)** Dice Similarity Coefficient (DSC). **(B)** Intersection over Union (IoU).

**Figure 8:**
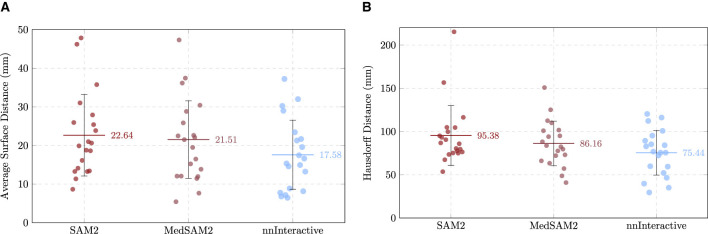
Comparison of the boundary metrics across the three models for each patient, with mean and standard deviation indicated. **(A)** Average Surface Distance (ASD). **(B)** Hausdorff Distance (HD).

**Figure 9:**
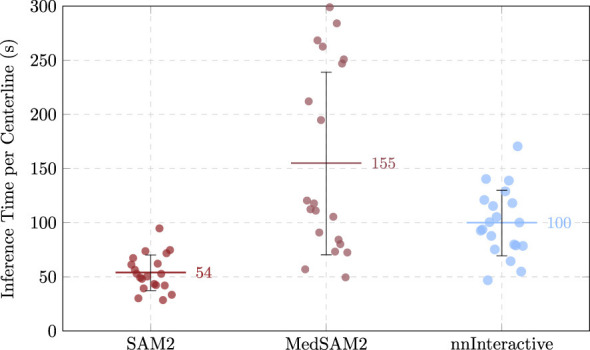
Comparison of the ITpC across the three models for each patient, with mean and standard deviation indicated.

Figures 7–9 show rain plots detailing the per-patient performance of the three models for volumetric overlap (DSC, IoU), boundary metrics (ASD, HD), and inference time per centerline (ITpC). These plots illustrate the variability across individual patients and highlight the advantage of nnInteractive.

To directly contrast the three models, Table 3 reports the mean and median values across all patients, and Supplementary Figures 11a–c illustrate a representative segmentation output from each one of the three models. Taken together, nnInteractive demonstrated the best balance of volumetric overlap, boundary fidelity, and efficiency. These results establish it as the most promising candidate for downstream fine-tuning, which was subsequently performed to assess the extent to which performance could be further optimized in this application.

**Table 3:** Zero-shot inference comparison of SAM2, MedSAM2, and nnInteractive.

**Metric**	**SAM2**	**MedSAM2**	**nnInteractive**
Mean (median) DSC	0.095 ± 0.066 (0.087)	0.075 ± 0.094 (0.037)	0.147 ± 0.095 (0.138)
Mean (median) IoU	0.051 ± 0.042 (0.045)	0.042 ± 0.056 (0.019)	0.082 ± 0.057 (0.074)
Mean (median) ASD (mm)	22.64 ± 10.55 (20.25)	21.51 ± 10.04 (20.49)	17.58 ± 8.95 (15.97)
Mean (median) HD (mm)	95.38 ± 34.60 (86.35)	86.16 ± 25.78 (83.06)	75.44 ± 25.87 (76.15)
Mean (median) ItpC (s)	53.73 ± 16.46 (51.55)	154.67 ± 84.27 (115.08)	99.60 ± 30.22 (96.96)

Metrics reported include DSC (Dice Similarity Coefficient), IoU (Intersection over Union), ASD (Average Surface Distance, mm), HD (Hausdorff Distance, mm), and ItpC (Inference time per centerline point, s). Values are presented as mean ± standard deviation with median in parentheses.

##### 3.1.4.1 Statistical comparison of zero-shot performance

The results of the Multivariate ANOVA test [Wilks' λ = 0.451, F_(10, 106)_ = 5.19, *p* < 0.001] confirmed that model choice had a significant multivariate effect on segmentation performance.

ANOVA tests on individual metrics revealed significant group differences for DSC [F_(2, 57)_ = 3.52, *p* = 0.036], IoU [F_(2, 57)_ = 3.17, *p* = 0.049], and ITpC [F_(2, 57)_ = 17.57, *p* < 0.001], while no significant differences were observed for HD (*p* = 0.115) or ASD (*p* = 0.293).

Tukey's pairwise comparisons further clarified these effects. For volumetric overlap, nnInteractive outperformed MedSAM-2 in both DSC (*p* = 0.034) and IoU (*p* = 0.049), while differences between SAM 2 and the other two models were not statistically significant. For computational efficiency, all three models differed significantly: SAM 2 was the fastest, followed by nnInteractive, and MedSAM-2, with all pairwise contrasts reaching significance (*p* ≤ 0.025). For a complete overview of the statistical analyses, including the ANOVAs F-values, p-values, and Tukey pairwise comparisons, see Supplementary Table S4.

Therefore, these statistical analyses corroborate the descriptive findings: although overall performance levels were low in the zero-shot setting, nnInteractive provided significantly better volumetric overlap than MedSAM-2 while maintaining a computational efficiency closer to SAM 2. This supports the selection of nnInteractive as the most promising candidate for downstream fine-tuning.

### 3.2 Impact of fine-tuning best-performing model

To further improve nnInteractive's segmentation performance, the model was fine-tuned on the subset of twenty cases, with the topology-aware compound SRL function. The process involved systematic exploration of training configurations, including patch and batch sizes, loss weighting strategies, and epoch scheduling, with the objective of identifying an optimal set of parameters for stable and accurate perforator segmentation. Further details on the hyperparameter optimization procedure are provided in Supplementary Section 4.4.

#### 3.2.1 Impact of fine-tuning

The impact of fine-tuning nnInteractive was evaluated on a held-out test set of ten previously unseen patients, using the same quantitative metrics as in the zero-shot experiments. The comparison demonstrated that fine-tuning led to improvements in segmentation accuracy. However, one patient (AVA070) was excluded from the analysis due to the fact that the fine-tuned model did not predict any foreground pixels. A retrospective review of AVA070 revealed that the perforator vessels in this volume were exceptionally small in caliber and appeared as a series of disconnected, high-intensity “islands” rather than a continuous tubular structure. While the zero-shot model captured these isolated voxels, the fine-tuned model, having learned stricter morphological and topological priors for continuous vascular structures, suppressed these disconnected regions as they did not meet the learned criteria for vessel continuity. This represents a trade-off where the model's increased robustness against noise in standard cases leads to the suppression of extremely fragmented vascular signals. For this reason, and to present a fair and direct comparison between models, the results from this patient were excluded from DSC, IoU, ASD, HD, and ITpC. The analysis proceeded with *n* = 9 patients.

Volumetric overlap showed a clear gain following fine-tuning. The mean DSC increased from 0.174 ± 0.127 (0.134) in the zero-shot setting to 0.265 ± 0.092 (0.298) post fine-tuning, representing an improvement of more than 50%. A similar trend was observed for the average IoU, which improved from 0.101 ± 0.084 (0.071) to 0.156 ± 0.059 (0.175), resulting in an increase larger than 1.5x. These results indicate that the fine-tuned model captured vessel regions more accurately and consistently than the baseline.

The mean ASD increased from 10.01 ± 4.03 (10.14) mm to 12.50 ± 7.50 (7.49) mm, while the mean HD saw an increase from 51.04 ± 9.17 (49.29) mm to 65.47 ± 18.60 (58.84) mm. This apparent deterioration in distance-based means warrants a more detailed investigation. In the zero-shot setting, predictions often consisted of small, isolated clusters near the vessel center; because these predictions were small and centrally located, their average and maximum distances to the ground truth surface remained mathematically low. In contrast, the fine-tuned model predicted more complete, elongated vascular trees. While these are more anatomically correct, any slight deviation in the trajectory of a long branch or the inclusion of a distal false-positive branch significantly penalizes the ASD and HD. Despite the increase in mean values, driven largely by outlier case AVA136 (ASD: 28.37 mm; HD: 99.55 mm), the median ASD decreased from 10.14 mm to 7.49 mm. This indicates that for the majority of patients, surface alignment actually improved. Crucially, the increased connectivity provided by fine-tuning was a prerequisite for the clinical analyses in Section 3.3. While the zero-shot masks (and the “decent” but fragmented predictions in cases like AVA070) were often too disconnected to allow for automated centerline extraction, the fine-tuned model provided the structural continuity necessary to calculate intramuscular path lengths. Inference times per centerline also saw a great decrease, with the fine-tuned model averaging ~35s compared to ~92s in the zero-shot setting. This indicates that the observed improvements in accuracy were achieved without compromising computational efficiency.

**Figure 10:**
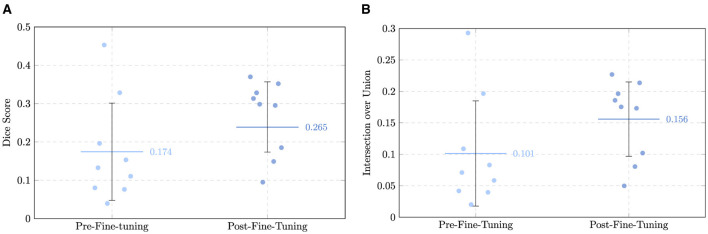
Comparison of overlap metrics across the two models for each patient, with mean and standard deviation indicated. **(A)** Dice Similarity Coefficient (DSC). **(B)** Intersection over Union (IoU).

**Figure 11:**
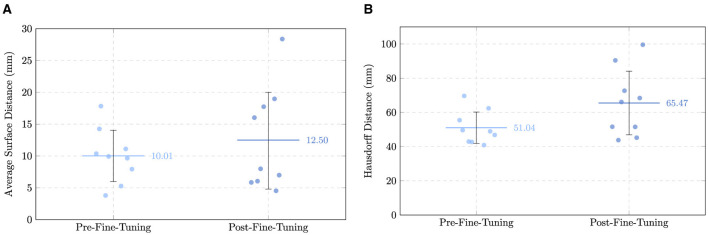
Comparison of boundary metrics across the two models for each patient, with mean and standard deviation indicated. **(A)** Average Surface Distance (ASD). **(B)** Hausdorff Distance (HD).

**Figure 12:**
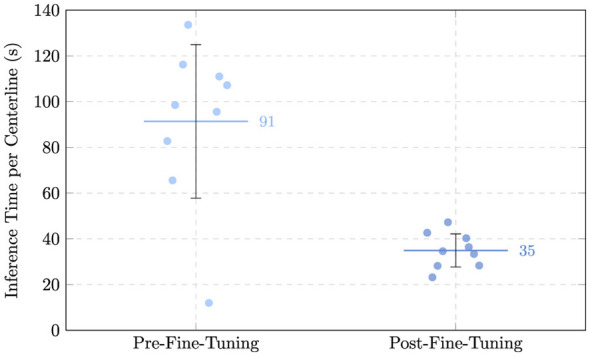
Comparison of ITpC across the two models for each patient, with mean and standard deviation indicated.

To directly contrast the three models, Table 4 reports the mean and median values across all metrics, while Figures 10–12 visualize scatter plots comparing metrics.

**Table 4:** Comparison of nnInteractive performance before and after fine-tuning.

**Metric**	**Pre-fine-tuning**	**Post-fine-tuning**
Mean (median) DSC	0.174 ± 0.127 (0.134)	0.265 ± 0.092 (0.298)
Mean (median) IoU	0.101 ± 0.084 (0.071)	0.156 ± 0.059 (0.175)
Mean (median) ASD (mm)	10.01 ± 4.03 (10.14)	12.50 ± 7.50 (7.49)
Mean (median) HD (mm)	51.04 ± 9.17 (49.29)	65.47 ± 18.60 (58.84)
Mean (median) ITpC (s)	91.36 ± 33.58 (98.5)	34.90 ± 7.25 (34.59)

Metrics reported include DSC (Dice Similarity Coefficient), IoU (Intersection over Union), ASD (Average Surface Distance, mm), HD (Hausdorff Distance, mm), and ItpC (Inference time per centerline point, s). Values are presented as mean ± standard deviation, with medians in parentheses.

**Figure 13:**
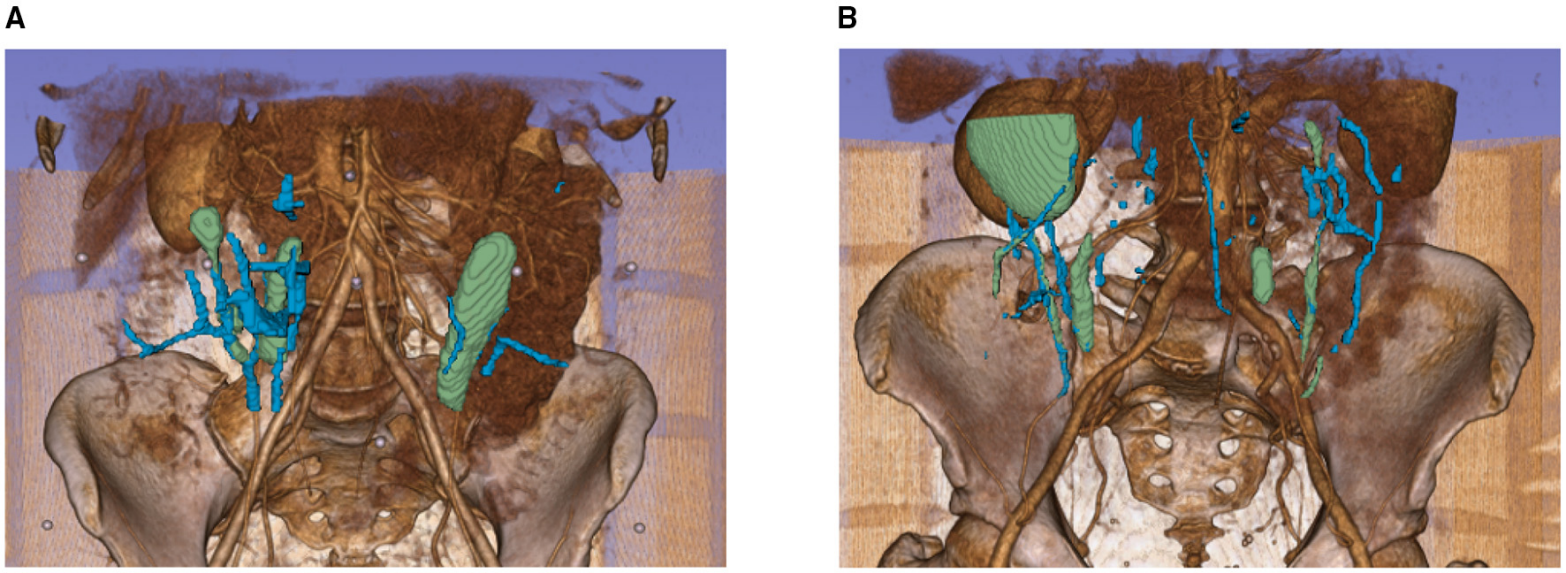
Example of perforator segmentation results before (green) and after (blue) fine-tuning, for patients **(A)** AVA116 and **(B)** AVA106.

Figure 13 shows the prediction results for two patients (AVA116 and AVA106). The qualitative inspection of these predictions further supports the quantitative improvements observed in the overlap-based metrics. In the pre-fine-tuning setting, the predicted segmentations often appeared as large, blob-like structures. While these predictions frequently encompassed the ground truth vessels, they exhibited substantial oversegmentation, leading to increased union area between ground truth and prediction. In contrast, the fine-tuned model produced predictions that were more anatomically plausible, with thinner and more elongated structures that more closely resembled vascular morphology. Although some degree of oversegmentation persisted, it was distributed in a manner that preserved vessel-like continuity rather than forming large spurious regions. While Figure 13 highlights cases (AVA116 and AVA106) where all metrics improved, we provide a balanced comparison in the Supplementary Figure S14, illustrating case AVA046, where the emergence of more complex vascular branches led to an increase in boundary-based error metrics despite better morphological representation. The pre–fine-tuned model's blob-like predictions tended to contain the ground truth but also included large volumes of false-positive tissue, inflating the predicted foreground and thus the union between prediction and ground truth. The fine-tuned model produced thinner, more vessel-like predictions whose foreground more closely matched the true vessel extent. Since both DSC and IoU quantify overlap relative to the union of prediction and ground truth, reducing large false-positive regions while preserving or modestly increasing the true overlap naturally led to higher values. In short, the move from blob-like to tubular predictions reduced spurious foreground volume and produced a union that better reflected the actual vessels, explaining the observed gains in both metrics.

On the other hand, the slight deterioration in boundary-based metrics, ASD and HD, can also be explained by this change in segmentation characteristics. By producing more elongated structures, the fine-tuned model occasionally introduced false-positive tubular branches in regions without corresponding ground truth, which can be observed in greater detail in Supplementary Figure S14. These spurious extensions increase the maximum distance between predicted and ground-truth surfaces (HD) and raise the overall surface discrepancy (ASD). The fact that median ASD improved while the mean worsened suggests that these errors were limited to a minority of cases, whereas most patients benefited from improved average surface alignment.

Overall, fine-tuning enhanced nnInteractive's performance by improving volumetric overlap with the ground truth on previously unseen data, producing more anatomically plausible vessel predictions. While boundary-based metrics showed a slight deterioration, likely due to occasional spurious branches introduced by the elongated predictions, the fine-tuned model still provided a more faithful representation of vascular morphology. These results confirm that adapting the model to the task-specific dataset yields meaningful gains, establishing the fine-tuned nnInteractive as the most robust foundation for the subsequent analyses.

### 3.3 Quantitative anatomical analysis of perforators

The quantitative results presented in this section are heavily dependent on the accuracy of the vessel segmentations produced by the fine-tuned model. Since both the intramuscular path length and the umbilicus distance metrics are derived directly from these segmentations, any imperfections can propagate into the downstream computations. Consequently, the interpretation of the errors reported here should be made considering that they may reflect not only limitations in the measurement procedures themselves but also the segmentation performance discussed in the preceding section.

#### 3.3.1 Intramuscular path length

The intramuscular length of the perforators, described in detail in Supplementary Table S10, was computed based on the centerlines extracted from the predicted vessel segmentations. For consistency, ground-truth intramuscular path lengths were derived using the same automated centerline extraction pipeline applied to the manual vessel segmentations, without manual correction of centerline paths at bifurcation points. This approach was chosen to ensure methodological symmetry between predicted and reference measurements and to avoid introducing observer-dependent bias in branch selection. The MAE between the predicted and ground-truth lengths was 11.59 mm (IQR: 4.19–14.37 mm), with a MdAE of 12.09 mm and a RMSE of 28.38 mm. Considering that both the MAE and MdAE are close to 12 mm (~1 cm, MdRAE < 30%), this error can be regarded as acceptable in relation to the anatomical scale of the vessels.

One likely source of error is the presence of bifurcations in the vessel paths. During centerline extraction, the algorithm may inadvertently switch branches at bifurcation points, creating sequences of points that “jump” between adjacent branches and thereby inflate the measured path length (Supplementary Figure S15). To investigate this hypothesis, a simplified metric was computed by measuring the straight-line distance between the start and end points of each centerline (Supplementary Table S11). Using this approach, the MAE decreased to 8.70 mm (IQR: 2.82–13.4 mm), the MdAE to 5.07 mm, and the RMSE to 12.24 mm, confirming that bifurcations were a major contributor to the overestimation of intramuscular lengths.

Additional factors may also play a role. The curvature of vessels is not always fully captured in the predicted segmentations, which can lead to slight under- or overestimation of the true path length. Furthermore, errors in the predicted segmentations themselves, such as truncated vessels, discontinuities, or spurious branches, can compound inaccuracies in centerline extraction and downstream intramuscular path length computation.

**Figure 14:**
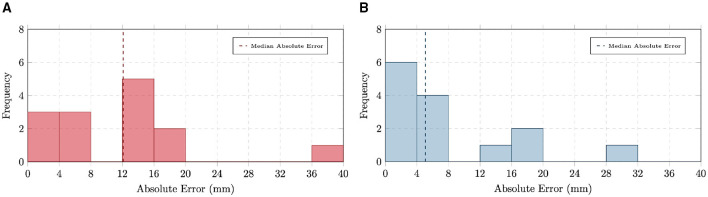
Distributions of absolute errors in intramuscular path length measurements using **(A)** centerline-based and **(B)** straight-line methods.

Figure 14 illustrates the absolute error distributions for the conventional centerline-based intramuscular path length (Figure 14A) and the straight-line path length (Figure 14B). These results highlight that while centerline-based metrics are valuable for detailed anatomical analysis, they remain sensitive to bifurcations and other structural complexities. Future improvements in centerline extraction algorithms, particularly in handling branch points, will be necessary to reduce these errors and achieve more reliable length measurements.

#### 3.3.2 Distance to umbilicus

**Figure 15:**
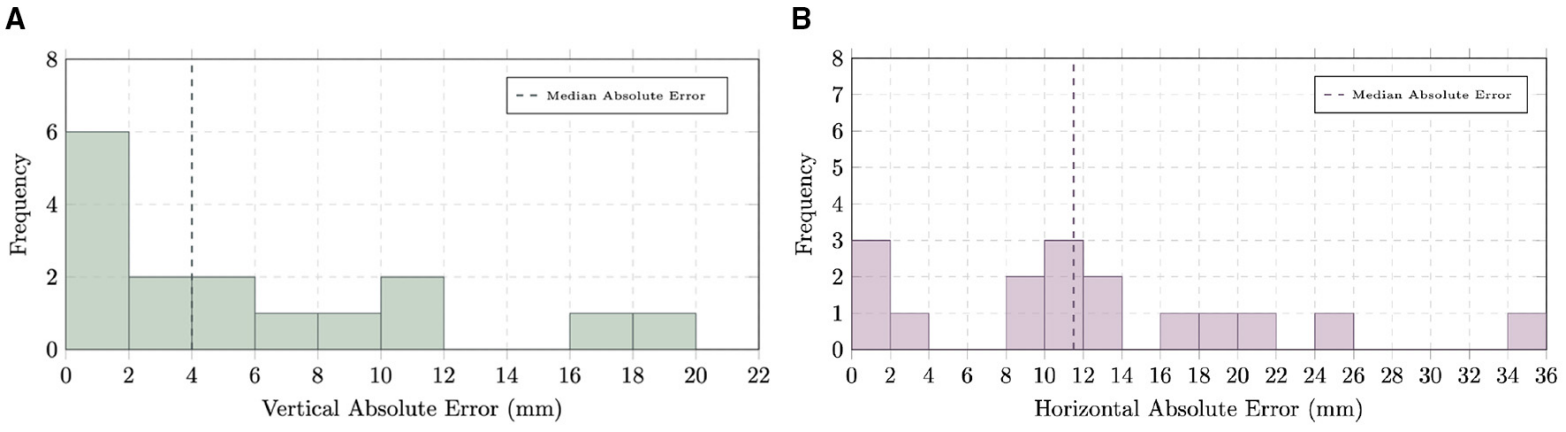
Distributions of absolute errors in perforator–umbilicus distance measurements along **(A)** vertical and **(B)** horizontal axes.

The algorithm identified an average of 2.6 perforator-fascia intersection points, which were points sufficiently close to the corresponding ground-truth locations reported in the radiology report, obtained to plan and guide surgery in these patients, across 6 of the 9 test patients. The distributions of absolute error along the vertical and horizontal axes are shown in Figure 15, with detailed values provided in Supplementary Tables S12, S13.

For the vertical axis, the MAE was 6.04 mm (IQR: 1–8.5 mm), with a MdAE of 4 mm and a RMSE of 8.36 mm. In contrast, the horizontal axis exhibited larger discrepancies, with an MAE of 12.51 mm (IQR: 7–17.5 mm), a MdAE of 11.15 mm, and a RMSE of 15.45 mm. These results indicate that prediction errors in the horizontal direction were substantially greater than those in the vertical direction.

A likely contributor to this discrepancy is imperfect umbilicus localization. In some cases, the umbilicus detection method failed to achieve totally accurate horizontal alignment, leading to systematic errors of a few millimeters. Consistent with this, the horizontal position of the umbilicus proved more difficult to predict than the vertical position, owing to anatomical variability and discrepancies in the regions of interest along the X axis. Another possible source of error lies in the course of the perforators themselves. Rather than crossing the fascia perpendicularly, many perforators traverse at an oblique angle, extending their crossing path over multiple sagittal slices. This angled trajectory complicates the precise determination of the fascia intersection point and may contribute additional uncertainty to the measurement of intramuscular lengths.

In addition, a substantial number of predicted perforators were truncated either within the muscle or near the fascia in the subcutaneous adipose tissue. While these predictions were sufficiently near the fascia to be paired with ground-truth perforators, they frequently lacked the distal segments present in the true anatomy. This systematic underrepresentation of the full perforator course contributed to inflated horizontal and vertical error values and reduced overall accuracy.

## 4 Discussion

This study demonstrates the feasibility of automating perforator vessel segmentation and anatomical measurement from CTA images for DIEAP flap breast reconstruction, addressing a critical bottleneck in current preoperative planning workflows. Prior research in autologous flap planning has predominantly focused on improving CTA acquisition protocols and manual or semi-automated tools (13, 14, 28, 29), leaving the task of fully automated perforator extraction largely unexplored. While deep learning has achieved major progress in vascular and organ segmentation (30–33), to the best of our knowledge, no prior work has systematically evaluated foundation models or topology-aware fine-tuning for perforator detection in DIEAP flap CTA. In this context, the present work introduces several novel contributions: a complete automated pipeline integrating tissue segmentation, centerline-based perforator extraction, benchmarking of promptable segmentation models, and automated computation of clinically relevant anatomical descriptors. Notably, the centerline extraction and prompting strategy were intentionally kept fixed throughout training and optimization. This design choice constrained the experimental space, allowing us to focus the evaluation on the adaptation behavior of foundation models under a consistent, clinically motivated prompting framework, rather than attempting to simultaneously optimize all pipeline components in this exploratory study. Consequently, the evaluation emphasizes the potential advantages of foundation models for perforator vessel segmentation in small datasets settings, rather than providing an exhaustive comparison with classical or vessel-specific methods that often require large datasets. Furthermore, and despite the performance, the proposed method allows an inital segmentation of the perforators that then can be used adjusted and corrected by the end-user, reducing the burden of a fully manual or a semi-automated tool.

Despite these advances, the study also faced important limitations that influence interpretation of the findings. The dataset was derived from a single center and included a limited number of CTA volumes, which constrained both fine-tuning and validation and precluded systematic evaluation of robustness across different scanners, acquisition protocols, or image-quality conditions. In addition, the present pipeline does not incorporate uncertainty estimation or automated failure-detection mechanisms, which are critical for assessing model confidence and ensuring clinical safety in real-world deployment.

The absence of publicly available annotated datasets required manual creation of a dedicated cohort, further limiting sample size. Heterogeneity in CT acquisition and contrast protocols introduced variability in perforator appearance, which likely contributed to errors in segmentation and, consequently, centerline extraction. Some modules of the computational pipeline, particularly the adapted umbilicus detection system and the centerline algorithm, showed inconsistent performance, especially in cases with vessel branching. Furthermore, explicit vascular topology enforcement (e.g., bifurcation-level consistency or graph-based constraints) is not implemented in the current approach; topology is only indirectly encouraged through loss design, which may have contributed to errors in vessel reconstruction. These factors collectively led to modest segmentation accuracy and highlight the inherent difficulty of modeling small, tortuous vascular structures under real-world imaging conditions. Another limitation is related to the isolation of contributions of key components (anatomical priors, prompting strategy, Skeleton Recall Loss, PMOO weighting) with ablation studies, which should be performed in a larger multi-centric study.

Even with these constraints, the results advance current understanding by demonstrating that a centerline-guided prompting strategy, combined with topology-aware loss functions, can improve the anatomical plausibility and continuity of perforator segmentations compared with zero-shot foundation model outputs. The automated derivation of intramuscular path length and umbilicus distance also illustrates how segmentation outputs can be translated into clinically actionable metrics, moving the field closer to automated, objective, and reproducible preoperative planning.

Future research should focus on expanding the dataset with additional CTA volumes acquired under standardized contrast and scanning protocols, enabling more robust model training and comparison. Synthetic vascular datasets could serve as an interim source for increasing data diversity. Technically, the pipeline would benefit from dedicated machine-learning methods for umbilicus detection and branching-aware centerline extraction capable of preserving vascular topology. Longer-term directions include developing specialized architectures tailored to different anatomical components and integrating the entire workflow into a clinically deployable system, including considerations for computational scalability, and PACS integration. Such advancements could substantially reduce the manual annotation burden, enhance the consistency of perforator selection, and support more reliable and efficient DIEAP flap planning.

## Data Availability

The original contributions presented in the study are publicly available. This data can be found here: https://github.com/saltapulga/AI-based-planning-for-DIEAP-flap-procedures.

## Footnotes

1 CompositIA (commit b053a0e), available at: https://github.com/rcabini/compositIA.

2 Skeleton Recall Loss (commit f259f63), available at: https://github.com/MIC-DKFZ/Skeleton-Recall.
