# Supplementary material for: AI-based planning for DIEAP flap procedures: exploring foundation models for artery perforators analysis
Source: Front Med (Lausanne). 2026 Mar 13;13:1757637. doi: 10.3389/fmed.2026.1757637 (PMC13021440; doi:10.3389/fmed.2026.1757637)
Supplement: Supplementary file 1 [file Data_Sheet_1.pdf]

Supplementary Material

1 DIEAP FLAP PREOPERATIVE PLANNING

Figure S1 below illustrates the manual CTA annotation process that allows for the computation of the perforators' caliber and distance to the umbilicus.

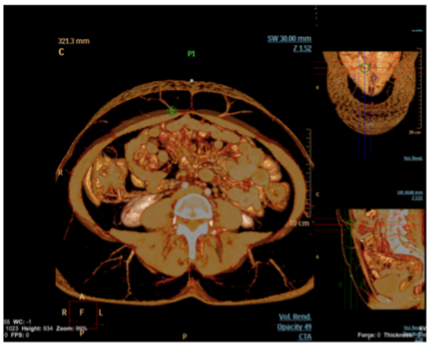

Figure S1a. Axial view

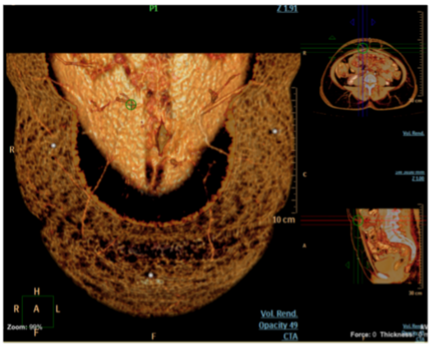

Figure S1b. Coronal view

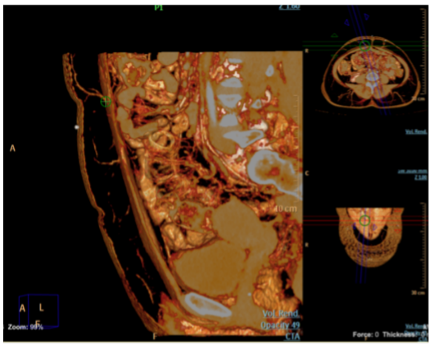

Figure S1c. Sagittal view

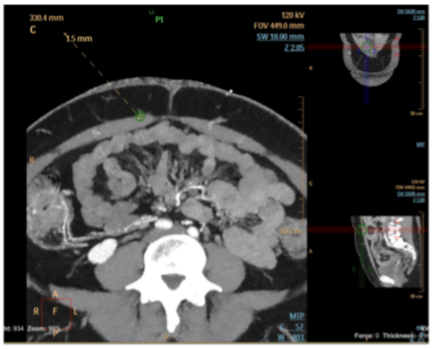

Figure S1d. Caliber computation

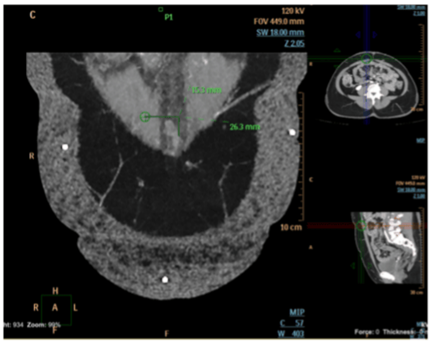

Figure S1e. Distance to the umbilicus computation

Figure S1. Fascial penetration mapping **a.-c.** and quantitative measurements **d.-e.** of a perforator.

2 GROUND TRUTH ANNOTATIONS SCHEMA

2.1 Segmentation 1 – Subcutaneous Perforators

The first segmentation, referred to as Segmentation 1, was dedicated to the annotation of subcutaneous perforator vessels. These annotations were performed on the standard CT volume (non-monoenergetic series). In this segmentation, Segment_1 was used to delineate the subcutaneous perforators, while Segment_2 represented the remaining background and surrounding anatomical structures.

2.2 Segmentation 2 – Intramuscular Perforators

The second segmentation, Segmentation 2, targeted the intramuscular perforator vessels. This segmentation was carried out on the MonoE40keV or MonoE45keV image series, depending on availability, which enhances the visualization of the muscular vascular paths due to their energy-specific attenuation profiles. Using the same labeling convention, Segment_1 was assigned to the intramuscular perforators,

while Segment_2 served as the non intramuscular perforator regions. The use of the monoenergetic reconstruction was critical to increase vessel prominence within the muscle tissue and reduce ambiguity in fine vascular structures.

2.3 Segmentation 3 – *Rectus Abdominis* Muscle

The third and final segmentation, Segmentation 3, was also performed on the MonoE40keV or MonoE45keV volume, focusing on delineating the *rectus abdominis* muscle. In this case, Segment_1 represented the muscle tissue, while Segment_2 again denoted the background.

2.4 File Formats, Organization, and Quality Control

All segmentations were saved in the *.seg.nrrd* format, and each was paired with its corresponding original volumetric CT image in *.nrrd format*. These files were organized within a standardized folder structure to ensure traceability and compatibility with downstream processing tools. Before finalizing each segmentation, annotations were reviewed and, when necessary, refined through consensus to guarantee consistency across the dataset. Particular attention was given to maintaining segmentation precision in regions of complex vascular bifurcation or ambiguous tissue contrast. Figure S2 illustrates the ground truth annotation results for one representative patient (AVA129), showcasing the subcutaneous perforators (blue), intramuscular perforators (green), and the *rectus abdominis* muscle (red) from anterior, posterior, and lateral views.

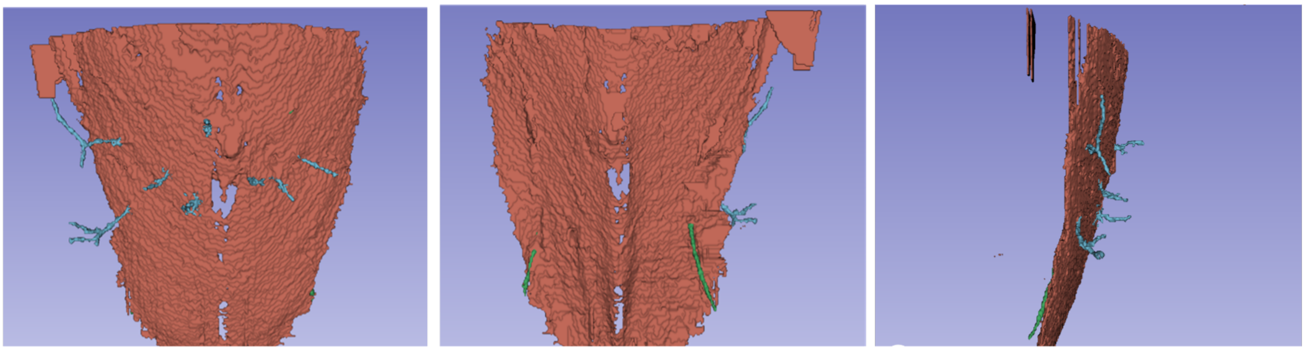

Figure S2a. **Figure S2b.** **Figure S2c.**

Figure S2. **(A)** Anterior, **(B)** Posterior and **(C)** Lateral view of ground truth annotation results for the subcutaneous (blue) and intramuscular perforators (green), and the muscle (red) of one patient (AVA129).

3 COMPUTER VISION ALGORITHM PIPELINE COMPONENTS

3.1 Maximum Intensity Projection

To mimic the MIP available in DICOM viewers and enhance algorithm performance by increasing brightness and contrast, the MIP algorithm was implemented. A slab thickness of 4 mm was selected, comprising 2 mm above and below each reference slice. Depending on the voxel spacing of the original volume, this translates to a projection over 4 to 8 slices per slab. This projection was applied in the axial plane, as it provides optimal visualization and alignment with anatomical references used during preoperative planning, such as the umbilicus.

However, MIP is limited by its nature as a 2D projection of 3D data. It inherently compresses depth information and can lead to an overestimation of vessel caliber, particularly in regions where overlapping structures are projected onto the same plane, or where vessels run obliquely to the axial plane, as their projected paths appear widened. Consequently, while the MIP image plays a crucial role in guiding centerline extraction, it is not used for final anatomical measurements, which rely instead on subsequent detailed segmentation from raw image data.

Additionally, the intensity-based nature of MIP reinforces the appearance of all bright and dense tissues, not only vessels, but also structures such as bone. This becomes a challenge when attempting segmentation based on global intensity thresholds, as these non-vascular elements may be mistakenly included. To mitigate this, the pipeline incorporates the definition of a RIO based on the umbilicus detection, to restrict the analysis to clinically meaningful areas and reduce false positives during centerline extraction.

The generation of MIP images successfully enhanced the visualization of contrast-enhanced structures. The resulting projections emphasized the bright signal of vascular pathways while suppressing much of the surrounding soft tissue, thereby facilitating the identification of candidate perforator trajectories. As shown in Figure S3, vessels appear as continuous bright paths across the abdominal wall, although the inherent projection effect resulted in visible overestimation of vessel caliber.

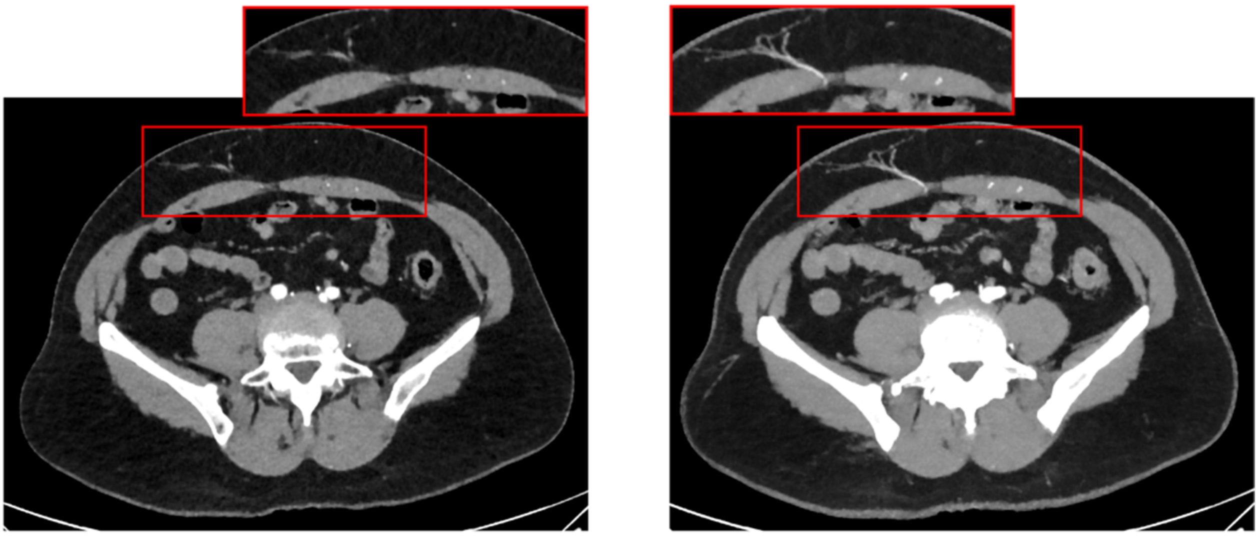

Figure S3a. **Figure S3b.**

Figure S3. **a.** Slice from raw CTA scan that originated the MIP image, **b.** MIP image highlighting enhanced visibility of vascular structures.

3.2 CompositIA – Subcutaneous and Visceral Adipose Tissue Segmentation

To segment the SAT, VAT, and SMA from the CT images, this study employed an adapted implementation of the CompositIA[1]. In this study, only the UNetL3 block was employed, focusing CompositIA's functionality toward the segmentation across the entire image volume, rather than being limited to the L3 slice.

Regarding the input data, before being fed into the model, each 2D axial CT slice was converted into a three-channel pseudo-RGB image. Each channel corresponded to a distinct HU window, tailored to

[1] CompositIA (commit b053a0e), available at: https://github.com/rcabini/compositIA

emphasize different tissue characteristics: the red channel was generated from the full spectrum (-1024 to 2048 HU), the green channel focused on the range typically associated with adipose tissue (-200 to -20 HU), and the blue channel was dedicated to the window commonly used for muscle tissue visualization (40 to 140 HU). This windowed strategy mimics radiological contrast adjustments, providing the model with tissue-specific intensity information and thereby enhancing class separability.

The normalized pseudo-RGB images were then processed by the CompositIA model, which produced a probability distribution across four classes: background, muscle (SMA), visceral fat (VAT), and subcutaneous fat (SAT). To obtain a final segmentation mask, these predictions were thresholded using class-specific confidence values. The thresholds were empirically determined to balance confident label assignment and false positive minimization. In particular, while the thresholds for SMA and VAT were reduced to 0.4 and 0.3, respectively, the SAT threshold was kept at 0.5 to ensure higher confidence in its classification. Notably, SAT was also the last label to be assigned during the post-processing step, meaning it could overwrite previous predictions in overlapping regions.

Post-processing steps involved removing a narrow margin along the lateral edges of each slice to minimize edge-related misclassifications, which can often arise from partial volume effects or abrupt transitions at the image boundaries. Additionally, the full 3D volume was reconstructed by stacking the individually segmented 2D predicted slices.

Although the CompositIA model provides a fast and effective way for labelling the major abdominal tissue compartments, its SMA segmentation lacks the spatial precision necessary for detailed anatomical analysis. To address this, a DL-based approach using TotalSegmentator was explored. The SAT mask produced by CompositIA remained an essential component of the pipeline. It played a critical role in delineating the region of interest for subcutaneous perforator centerline extraction, restricting candidate detections to the anterior SAT region and thereby reducing the likelihood of false positives in unrelated tissues such as intestinal loops or bone. Thus, even with the transition to TotalSegmentator for muscle segmentation, the CompositIA output continued to provide vital anatomical context for the subcutaneous domain.

The CompositIA model provided a fast and effective delineation of SAT, VAT, and SMA compartments.

The output 3D masks, represented as 2D slices in Figure S4, demonstrate the accurate separation of SAT and VAT regions, enabling the definition of regions of interest for downstream perforator analysis. However, as anticipated from the methodological description, SMA segmentation lacked spatial precision, with the muscle label erroneously including, in some cases, portions of the perforators located within the subcutaneous adipose tissue.

3.3 Skin Segmentation

In parallel with the application of the CompositIA model, an additional segmentation step was implemented to extract the skin surface. This step was essential for accurately localizing the external contours of the body, which plays a critical role in subsequent processes, most notably the detection of the umbilicus. Furthermore, the resulting skin mask was also leveraged to support the refinement of the *rectus abdominis* muscle segmentation by providing anatomically meaningful spatial boundaries.

The skin mask was generated through a straightforward intensity-based thresholding operation applied to the original image volume, with HU values constrained between a lower threshold of -400 HU and an upper threshold of 1500 HU. These thresholds were chosen to broadly encompass all soft and hard tissue but exclude the background. However, to isolate the outermost skin layer with greater specificity, a multi-step

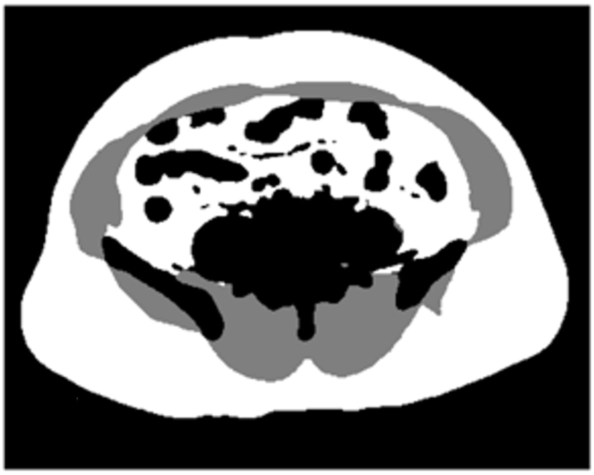

Figure S4a.

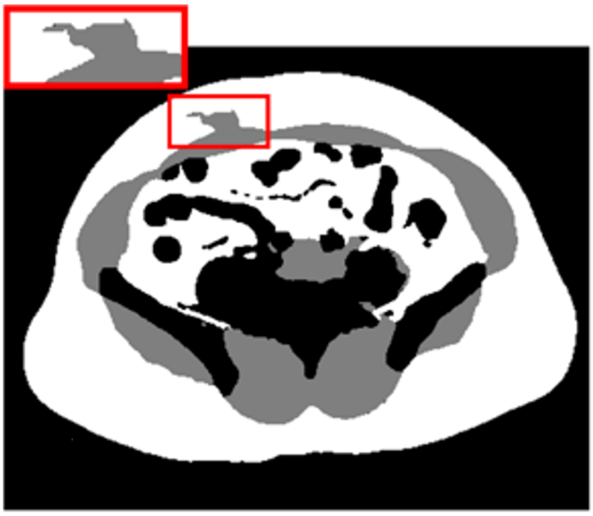

Figure S4b.

Figure S4. **a.** Optimal prediction of SAT, VAT, and SMA by CompositIA, **b.** Suboptimal prediction of SMA by CompositIA.

morphological post-processing procedure was required. This process began with a morphological opening to eliminate small external structures, followed by the extraction of the largest connected component to retain the primary body outline. Subsequently, a binary fill operation was performed to close internal holes within the component. An erosion step was then applied to contract the filled region slightly, and the final skin outline mask was obtained by subtracting the eroded mask from the filled one. This series of operations effectively produced a thin shell corresponding to the external skin surface.

All morphological operations that required a structuring element were performed using a spherical kernel ($sitk.sitkBall$) to ensure isotropic behaviour across all dimensions. The kernel radii used for each operation were defined as follows: **Morphological Opening**: $[4, 4, 4]$; **Erosion:** $[5, 5, 5]$.

The skin segmentation algorithm yielded a thin, continuous mask encasing the entire volume, providing a reliable outer boundary later used to refine the muscle segmentation. This thin contour successfully excluded internal tissues and was robust across cases, showing minimal discontinuities or gaps. Figure S5 presents the output of some steps of the skin segmentation pipeline.

3.4 Umbilicus Detection Algorithm Implementation and Results

3.4.1 Overview

In abdominal image analysis, the umbilicus serves as a consistent and anatomically significant reference point. Its identification is particularly valuable in scenarios where precise localization of muscle groups, such as the *rectus abdominis*, or vascular structures, is required. Given its central location along the anterior abdominal wall, the umbilicus serves as a reliable landmark to define ROIs in a generalized, yet subject-specific, manner. Recognizing its importance, a dedicated algorithm was developed as part of the proposed pipeline to automatically detect the umbilicus based on the skin's surface anatomy extracted from the CT volume. This approach was implemented using a combination of morphological operations and contour analysis techniques applied to the previously segmented skin mask. Figure S6 illustrates the pipeline used in this algorithm.

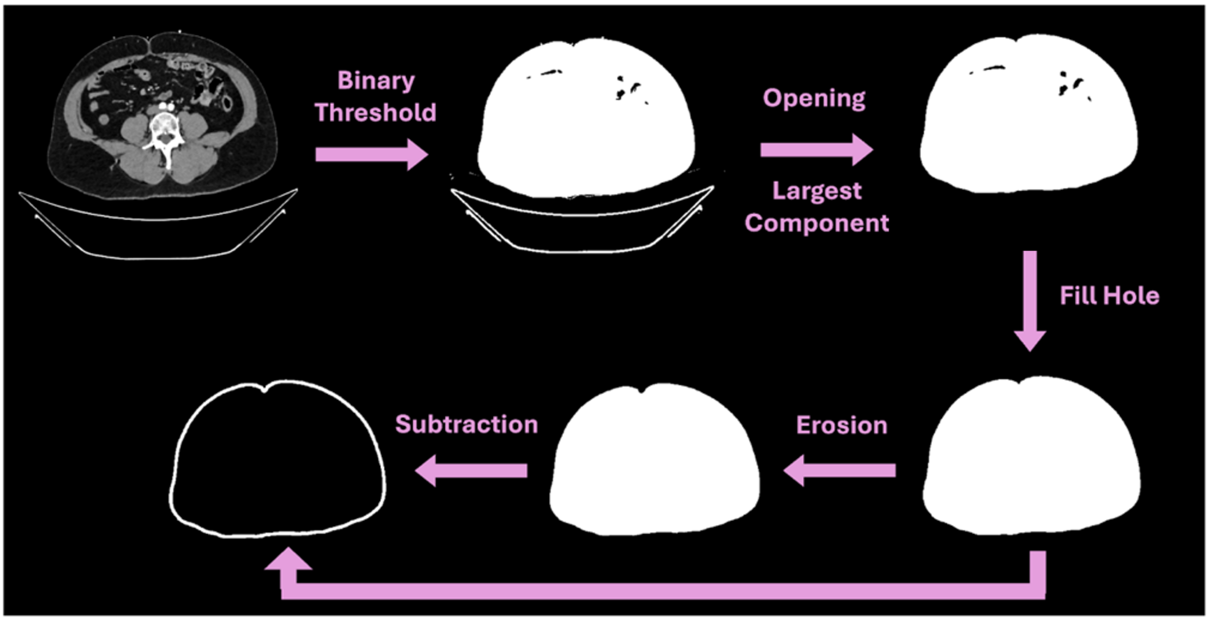

Figure S5. Skin segmentation algorithm.

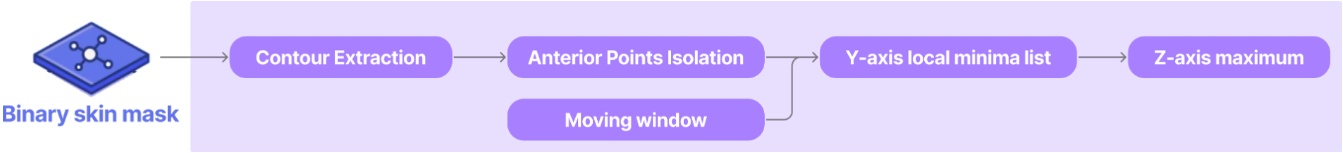

Figure S6. Overview of the Umbilicus Detection Algorithm

In short, the algorithm analyzes contours of the segmented skin mask to isolate the anterior surface and identify local minima corresponding to the umbilical depression. Candidate points are aggregated across axial slices, and the final umbilical coordinate is selected based on its axial position. This 3D point provides a stable anatomical anchor for subsequent segmentation of abdominal muscles and fascia, as well as for quantitative assessments such as computing distances to perforator vessels. The algorithm identifies the umbilicus by analyzing anterior body contours extracted from axial slices of a binary skin mask. Key steps include:

- **Contour extraction** - the outermost connected component is identified in each slice using `cv2.findContours`, focusing on 50% to 75% of the volume depth-wise.
- **Anterior point isolation** - filtering contour points to retain only those in the anterior region of the body.
- **Local minima detection** - identifying concave depressions along the anterior contour representing potential umbilicus points.
- **Candidate point selection** - averaging multiple local minima per slice to produce a single representative point.
- **Final point determination** - selecting the candidate with the highest z-value as the most likely umbilicus position.

3.4.2 Preprocessing and Contour Extraction

Contours are extracted from each axial slice of the binary skin mask. Only the outermost connected component is considered. To reduce computational load and improve robustness, this analysis is restricted to slices between 50% and 75% of the volume depth-wise, capturing the anatomical region where the umbilicus typically resides.

An example of the extracted outer contour is shown in Figure S7a., where the red outline represents the detected body surface.

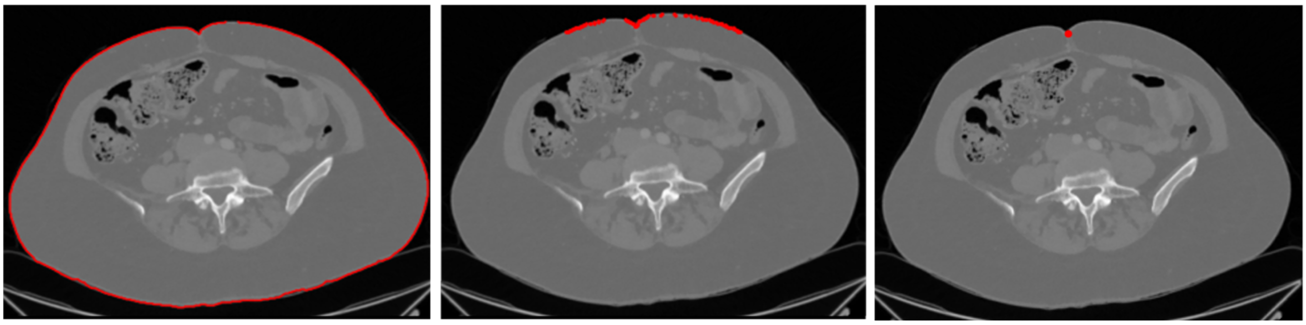

Figure S7a. **Figure S7b.** **Figure S7c.**

Figure S7. **a.** Example of contour extraction step from the binary skin mask. The outermost connected component (red outline) represents the body surface contour, **b.** Detection of anterior local minima along the extracted contour. Candidate minima (red dots) highlight concave depressions in the anterior abdominal surface, **c.** Final umbilicus detected point.

3.4.3 Anterior Point Isolation and Local Minima Detection

The anterior region of the body is defined by the upper half of y-coordinates and the central third of x-coordinates. Contour points in this region are examined for local minima along the y-axis. Each point is compared with its neighbors in a moving window, and consistent minima are stored as potential umbilicus points. If multiple minima exist in a slice, their coordinates are averaged (median of x and y values) to reduce sensitivity to noise.

Figure S7b. illustrates this step, where local depressions in the anterior abdominal surface are automatically highlighted as candidate umbilicus locations.

3.4.4 Candidate Point Selection and Final Determination

All candidate umbilicus points are stored as 3D coordinates (z, x, y). The point with the highest z-coordinate (lowest axial position) is selected as the final umbilicus point, as shown in FigureS7c.. This point serves both as an anatomical landmark and as a reference for downstream processing, including defining the ROI for abdominal muscle segmentation and computing Euclidean distances to perforator vessel intersections with the fascia.

3.5 TotalSegmentator-Based Muscle Segmentation

The integration of the TotalSegmentator[2] model into this study was made possible by the recent release of additional license-free segmentation tasks provided by the model's developers. However, it is important

[2] TotalSegmentator (commit e69a31a), available at: https://github.com/wasserth/TotalSegmentator

to acknowledge that these license-free models were trained on a limited dataset, primarily focusing on the anatomical region extending from the T4 to the L4, as specified by the developers.

The decision to adopt the TotalSegmentator-based segmentation was driven by its markedly improved anatomical accuracy and reduced noise relative to the earlier CV-based approach. These enhancements significantly benefited downstream analyses, such as perforator vessel centerline extraction, which are particularly sensitive to segmentation quality.

The abdominal muscle segmentation produced by TotalSegmentator included the *pectoralis*, *serratus*, external and internal obliques, *psoas*, *trapezius*, *quadratus lumborum*, *latissimus dorsi*, *transversospinalis*, *erector spinae*, as well as the left and right *rectus abdominis* muscles. These last two individual muscle labels were subsequently merged to form a unified mask that defined the ROI within the original CT images for subsequent perforator vessel analysis. The segmentation of the *rectus abdominis* muscles using TotalSegmentator produced accurate and anatomically consistent masks, as illustrated in Figure S8. As anticipated from the methodological description, the segmentations were cut short at the inferior limit, since the model was trained only to segment structures within the T4–L4 vertebral range.

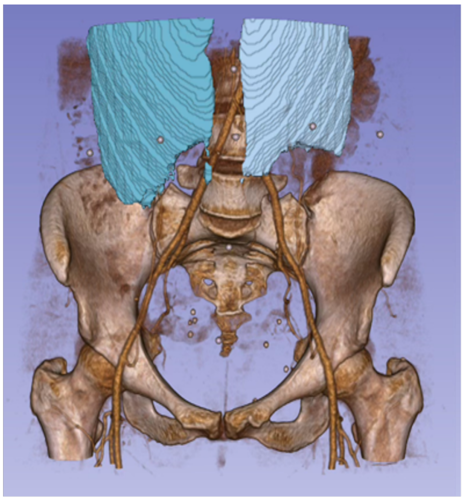

Figure S8a.

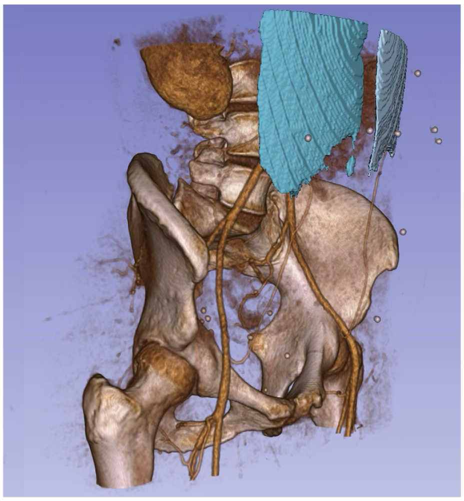

Figure S8b.

Figure S8. **a.** Anterior and **b.** Lateral view of the TotalSegmentator *rectus abdominis* right (darker blue) and left (lighter blue) prediction results.

3.6 Intramuscular and Subcutaneous Perforator Segmentations

With the anatomical layers segmented, the subsequent step focused on isolating perforator vessels transversing either the abdominal muscle or the subcutaneous tissue.

Intramuscular perforators were identified from the MIP of the original CT, previously masked to retain only the muscle tissue. A binary threshold defining the foreground to be between 85 and 350 HU was applied, followed by a morphological closing operation and connected component analysis. Objects with a volume below 200 voxels were discarded to eliminate small non-vascular structures.

For the subcutaneous perforators, segmentation began using the previously computed SAT mask. A binary threshold between 1 and 350 HU was applied to the eroded subcutaneous layer to isolate voxels within the intensity range typically associated with vascular structures in fat. Given the higher contrast between subcutaneous perforators and the surrounding adipose tissue, this segmentation task was inherently

simpler than its intramuscular counterpart. To further enhance anatomical continuity, a morphological closing operation with a spherical kernel, with radius $[2, 2, 2]$ voxels, was applied. Finally, connected component analysis was performed, and components smaller than 50 voxels were excluded. This process produced a spatially localized binary mask of candidate subcutaneous perforators in the periumbilical region. Using the muscle and adipose tissue masks, binary thresholding and morphological refinement steps successfully isolated candidate perforators. As expected and illustrated in FigureS9, intramuscular perforator (red) detection was more challenging, producing fewer and sometimes enlarged candidates, while subcutaneous perforators (green) appeared more clearly within the SAT mask due to their higher contrast with surrounding fat tissue.

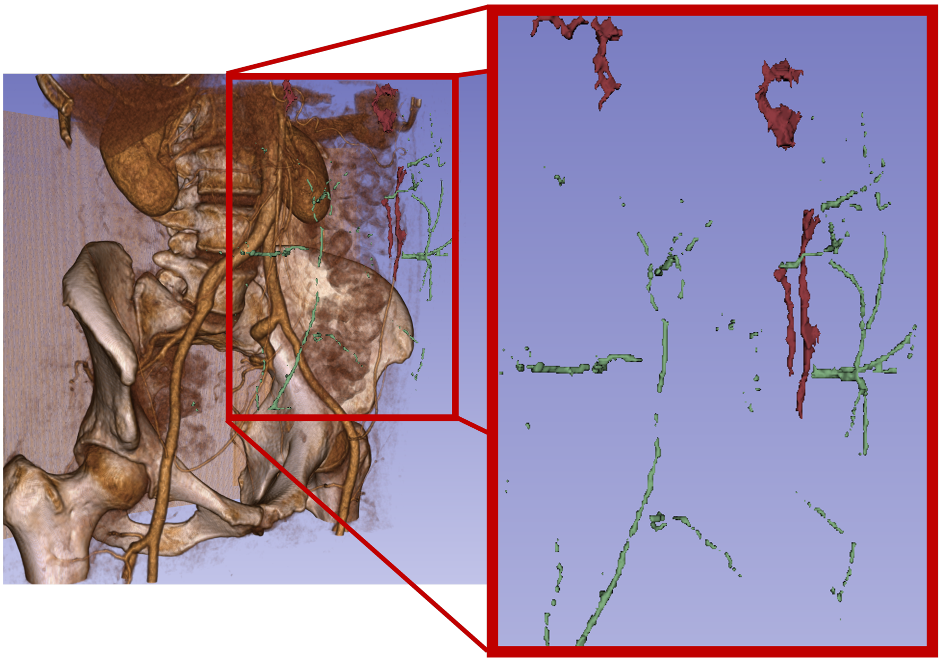

Figure S9. Intramuscular (red) and subcutaneous (green) perforator cv-based segmentation results.

3.7 Centerline Extraction Algorithm and Prompting Strategy

3.7.1 Overview

The algorithm generates centerlines for perforator vessels by calculating the 2D center of mass of segmented pixels in each axial slice of a labelled segmentation map. Each connected vessel is assigned a unique label, and points are stacked across slices to form the centerline trajectory. This representation serves as a structural prior for downstream vessel segmentation.

3.7.2 Centerline Extraction Procedure

For each labelled object:

- Axial slices containing segmented pixels are identified.
- The 2D center of mass is computed on each slice, producing a single representative point per slice.
- Stacking these points across slices forms an approximate centerline trajectory.

Limitations of this approach include potential inaccuracies at bifurcations, where the center of mass may fall between branches, deviating from the true lumen path.

3.7.3 Spatial Transformation and Filtering

Extracted voxel-based indices are transformed into real-world spatial coordinates using the `TransformContinuousIndexToPhysicalPoint` function from SimpleITK, with direction cosines adjusted to the Left, Posterior, Superior (LPS) coordinate system. Only centerlines containing more than ten points are retained to remove spurious detections. Each accepted centerline is serialized into a JSON file compatible with 3D Slicer, including spatial coordinates and metadata for visualization.

3.7.4 Ranking and Downstream Use

Centerlines are ranked by axial extent to filter noisy or fragmented detections, under the assumption that valid vascular structures span multiple slices. Filtered centerlines are passed to the deep learning-based segmentation module, serving as structural priors to guide fine-grained vessel delineation.

3.7.5 Results from This Study

From the segmented perforators, centerlines were extracted as markup points. This process generated ordered sets of points along each vessel, representing its approximate trajectory in 3D space, shown in Figure S10. These centerlines provided interpretable anatomical guidance and were subsequently used as structural priors in the DL-based segmentation stage. Although deviations occurred in regions with bifurcated courses, the extracted markup points overall aligned well with the segmented perforator paths.

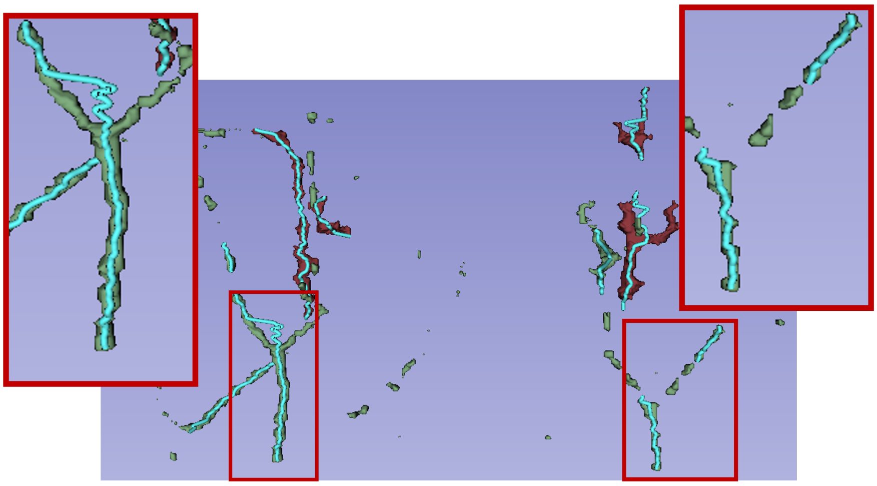

Figure S10. Example of centerline extraction from perforator segmentations, showing correctly extracted centerlines(right), and the bifurcation issue (left).

3.7.6 Limitations and Corrective Strategy

While the method may deviate in complex regions such as bifurcations, it provides a valuable intermediate representation. In the segmentation stage, bright voxels near each centerline point are evaluated to refine the estimated vessel path, partially compensating for center-of-mass deviations and increasing robustness to branching complexity.

4 MODEL COMPARISON AND ZERO-SHOT BENCHMARKING

4.1 SAM 2, MedSAM-2, and nnInteractive Model Results

Tables S1, S2, and S3 present the patient-specific results for SAM 2, MedSAM-2, and nnInteractive performance on the zero-shot benchmarking, respectively.

Table S1. Patient-level DSC, IoU, ASD, HD, HD95, and ITpC values for SAM 2.

Case	DSC	IoU	ASD (mm)	HD (mm)	ItpC (s)
ART2394	0.004	0.002	11.35	53.58	52.82
AVA100	0.102	0.054	27.90	85.87	41.97
AVA101	0.087	0.045	19.88	90.55	48.17
AVA102	0.087	0.045	13.24	215.32	42.34
AVA104	0.129	0.069	8.65	95.06	61.24
AVA107	0.073	0.038	35.75	116.39	33.43
AVA108	0.001	0.001	47.83	99.76	73.73
AVA109	0.344	0.208	13.39	80.41	94.70
AVA110	0.097	0.051	13.26	93.27	56.46
AVA112	0.123	0.066	25.39	79.66	71.78
AVA114	0.117	0.062	14.10	67.36	49.28
AVA118	0.112	0.059	20.62	74.94	28.53
AVA124	0.071	0.037	16.13	73.48	50.31
AVA129	0.039	0.020	18.77	75.16	43.37
AVA133	0.046	0.024	46.20	156.61	30.15
AVA139	0.045	0.023	23.84	76.35	74.72
AVA148	0.066	0.034	31.01	104.84	39.30
AVA149	0.086	0.045	20.97	104.58	62.15
AVA150	0.156	0.085	25.93	86.83	67.33
AVA151	0.114	0.060	18.61	77.66	52.78
**Mean**	0.095 ± 0.066	0.051 ± 0.042	22.64 ± 10.55	95.38 ± 34.60	53.73 ± 16.46
**Median**	0.087	0.045	20.25	86.35	51.55
**Range**	[0.001, 0.345]	[0.001, 0.208]	[8.65, 47.83]	[53.58, 215.32]	[28.53, 94.70]
**Mean HD95 (Median)**				59.64 ± 22.29 (55.55)	

4.2 Comparative Analysis

Figure S11 illustrates representative segmentation outputs from the three evaluated models: SAM 2, MedSAM-2, and nnInteractive, respectively. Each subfigure displays the predicted segmentation overlayed on the original CT slice. These examples highlight the qualitative differences in segmentation accuracy and boundary delineation among the models.

For completeness, Figure S12 presents the grouped bar plots of average zero-shot performance metrics for SAM2, MedSAM2, and nnInteractive. These plots summarize the mean and standard deviation across all patients for volumetric overlap (DSC, IoU) and boundary accuracy (ASD, HD). While the main Results

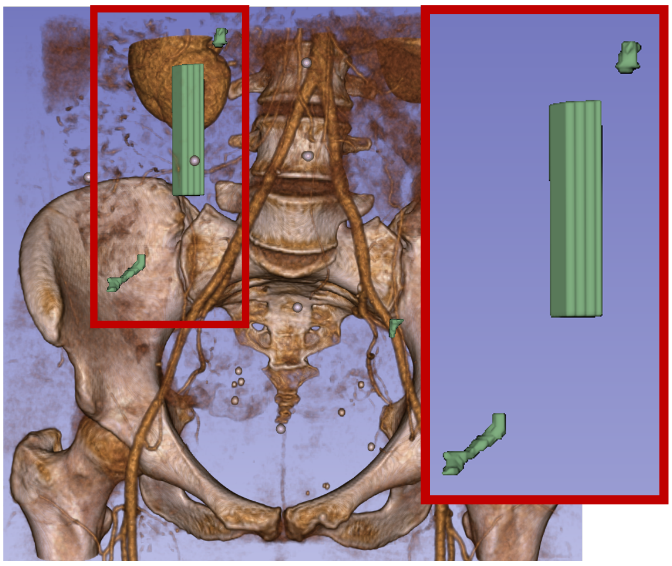

Figure S11a.

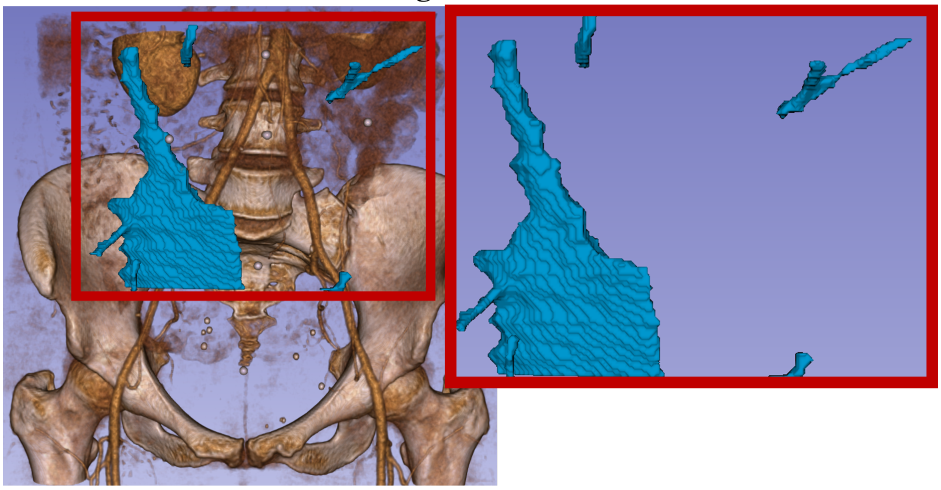

Figure S11b.

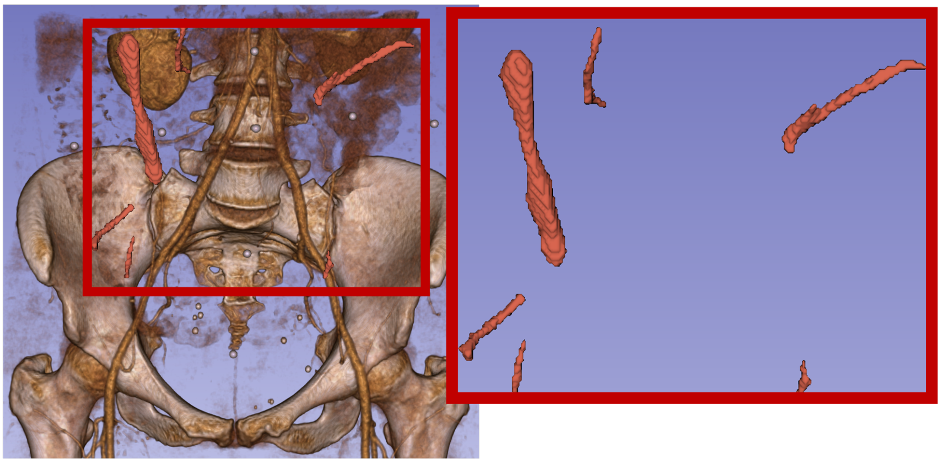

Figure S11c.

Figure S11. **a.** Example of SAM 2 model predictions, **b.** Example MedSAM-2 model predictions, **c.** Example nnInteractive model predictions.

Table S2. Patient-level DSC, IoU, ASD, HD, HD95, and ITpC values for MedSAM-2.

Case	DSC	IoU	ASD (mm)	HD (mm)	ItpC (s)
ART2394	0.011	0.006	25.84	94.05	268.35
AVA100	0.113	0.060	16.47	82.36	84.29
AVA101	0.049	0.025	12.06	88.15	120.44
AVA102	0.019	0.010	13.84	40.93	72.48
AVA104	0.042	0.021	12.06	83.77	117.77
AVA107	0.025	0.013	22.62	101.71	73.26
AVA108	0.014	0.007	37.43	125.14	194.78
AVA109	0.371	0.228	11.94	79.59	246.95
AVA110	0.004	0.002	47.31	150.83	112.38
AVA112	0.227	0.128	19.48	77.63	105.45
AVA114	0.028	0.014	5.42	66.08	56.93
AVA118	0.005	0.003	30.41	73.05	49.52
AVA124	0.009	0.004	21.50	63.32	90.92
AVA129	0.042	0.022	11.45	48.74	80.16
AVA133	0.052	0.027	36.15	112.53	111.10
AVA139	0.023	0.011	15.22	72.09	298.98
AVA148	0.031	0.016	22.47	100.92	212.04
AVA149	0.082	0.043	22.06	95.03	284.09
AVA150	0.126	0.067	28.81	110.03	262.63
AVA151	0.223	0.125	7.64	57.17	250.80
**Mean**	0.075 ± 0.094	0.042 ± 0.056	21.51 ± 10.04	86.16 ± 25.78	154.67 ± 84.27
**Median**	0.037	0.019	20.49	83.06	115.08
**Range**	[0.004, 0.371]	[0.002, 0.228]	[7.64, 47.31]	[40.93, 150.83]	[49.52, 298.98]
**Mean HD95 (Median)**				60.93 ± 25.99 (54.27)	

section emphasizes per-patient variability using rain plots, this figure provides an overview of the models' aggregated performance for readers interested in average trends.

4.3 Statistical Testing Results

This section presents the statistical analysis performed to evaluate the significance of performance differences among the three zero-shot segmentation models (SAM 2, MedSAM-2, and nnInteractive). One-way ANOVA was applied to each evaluation metric to assess whether mean differences across models were statistically significant. Where ANOVA indicated significance, Tukey's Honest Significant Difference (HSD) test was conducted to identify specific pairwise differences between models. Results are presented in Table S4.

4.4 Fine-Tuning Hyperparameter Optimization

The impact of different patch and batch size configurations was first evaluated, with the predefined setup of $[96, 160, 160]$ and batch size 2 serving as the reference point. A series of alternative combinations was tested over 30 epochs (Table S5). Mean validation dice (MVD) scores ranged from 0.464 to 0.533, with the

Table S3. Patient-level DSC, IoU, ASD, HD, HD95, and ITpC values for nnInteractive

Case	DSC	IoU	ASD (mm)	HD (mm)	ItpC (s)
ART2394	0.093	0.059	7.15	29.51	100.52
AVA100	0.250	0.143	21.67	84.18	78.92
AVA101	0.348	0.211	6.80	89.31	93.79
AVA102	0.017	0.009	13.23	35.08	78.55
AVA104	0.317	0.188	6.45	85.26	115.39
AVA107	0.260	0.149	31.99	116.18	64.42
AVA108	0.016	0.008	28.99	120.33	140.33
AVA109	0.217	0.122	19.58	75.54	170.52
AVA110	0.062	0.032	8.86	76.76	105.26
AVA112	0.151	0.082	21.28	75.51	138.87
AVA114	0.137	0.074	7.75	39.74	92.63
AVA118	0.209	0.117	16.61	59.40	54.86
AVA124	0.215	0.120	15.33	59.83	87.79
AVA129	0.112	0.059	14.93	52.12	79.88
AVA133	0.055	0.028	37.22	112.27	46.83
AVA139	0.139	0.075	23.41	72.34	128.95
AVA148	0.025	0.013	14.58	95.25	75.30
AVA149	0.089	0.047	17.45	100.74	118.07
AVA150	0.088	0.046	30.24	82.86	121.00
AVA151	0.142	0.076	8.15	46.55	100.12
**Mean**	0.147 ± 0.095	0.082 ± 0.057	17.58 ± 8.95	75.44 ± 25.87	99.60 ± 30.22
**Median**	0.138	0.074	15.97	76.15	96.96
**Range**	[0.016, 0.348]	[0.008, 0.211]	[6.45, 37.22]	[29.51, 120.33]	[46.83, 170.52]
**Mean HD95**				50.14 ± 25.89 (44.54)	

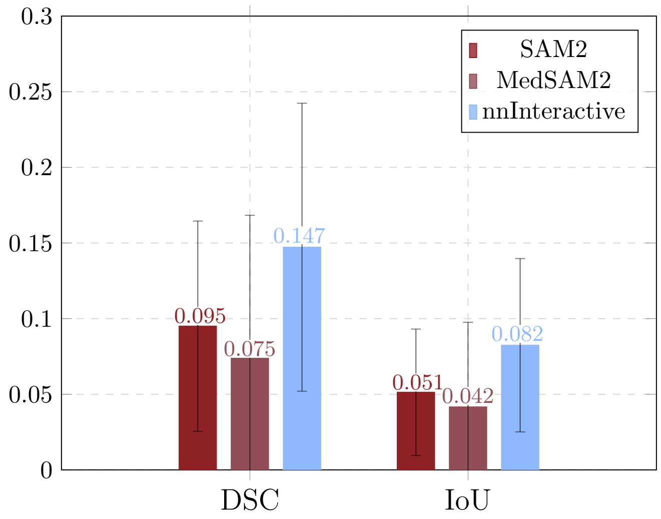

Figure S12a.

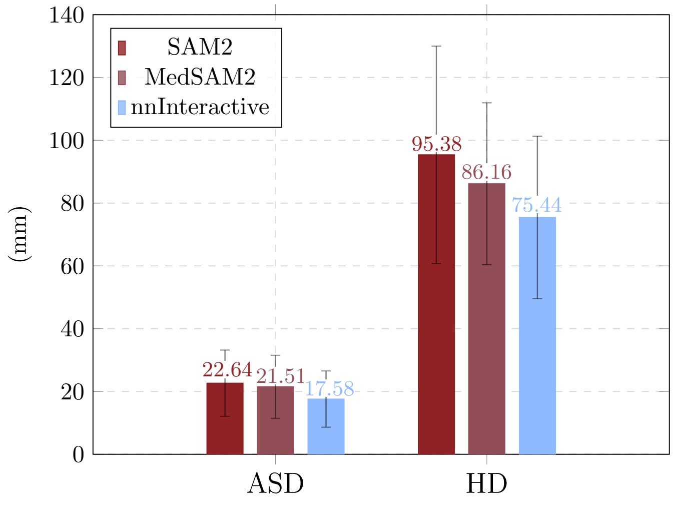

Figure S12a.

Figure S12. **a.** Comparison of averaged overlap-based metrics (DSC and IoU) and **b.** Comparison of averaged boundary-based metrics (ASD and HD) across the three state-of-the-art image segmentation foundation models, with mean and standard deviation indicated.

Table S4. ANOVA and Tukey HSD Results of Zero-Shot Model Performance

Metric	ANOVA F (df)	p-value	Significant	Tukey Pairwise Comparisons	Significant
**DSC**	3.52 (2,57)	0.036	Yes	MedSAM2 vs SAM2: 0.756<br>MedSAM2 vs nnInteractive: 0.034<br>SAM2 vs nnInteractive: 0.161	MedSAM2 vs nnInteractive
**IoU**	3.17 (2,57)	0.049	Yes	MedSAM2 vs SAM2: 0.832<br>MedSAM2 vs nnInteractive: 0.049<br>SAM2 vs nnInteractive: 0.167	MedSAM2 vs nnInteractive
**ASD**	1.25 (2,57)	0.293	No	MedSAM2 vs SAM2: 0.871<br>MedSAM2 vs nnInteractive: 0.548<br>SAM2 vs nnInteractive: 0.275	None
**HD**	2.24 (2,57)	0.115	No	MedSAM2 vs SAM2: 0.593<br>MedSAM2 vs nnInteractive: 0.495<br>SAM2 vs nnInteractive: 0.096	None
**ItpC**	17.57 (2,57)	< 0.001	Yes	MedSAM2 vs SAM2: 0.000<br>MedSAM2 vs nnInteractive: 0.006<br>SAM2 vs nnInteractive: 0.025	All

best performance achieved for patch size [96, 192, 192] and batch size 4. This combination was therefore selected for subsequent experiments.

Table S5. Batch and Patch Size Optimization

Name	Patch Size	Batch Size	Average Epoch Time (s)	Mean Validation Dice
COMBO1.1	[96,160,160]	2	82.9	0.465
COMBO1.2	[128,192,192]	2	140.37	0.520
COMBO1.3	[96,192,192]	2	110.63	0.487
COMBO1.4	[128,128,128]	2	73.07	0.486
COMBO1.5	[96,160,160]	4	146.07	0.517
COMBO1.6	[128,192,192]	4	262.93	0.484
COMBO1.7	[96,192,192]	4	202.07	**0.533**
COMBO1.8	[128,128,128]	4	127.53	0.492

During the next stage, the relative contributions of the different loss terms were adjusted. It was observed that the CE loss was consistently much smaller in magnitude than Dice and SRec losses. To counteract this imbalance, different weight combinations of Dice, SRec, and CE were tested. When using equal weighting (Dice: 0.33, SRec: 0.33, CE: 0.33), a MVD of 0.533 was achieved, but loss evolution was suboptimal. Increasing the weight of Dice and SRec (decreasing CE) to [0.4, 0.4, 0.2] lowered the MVD to 0.499.

In addition to some manual experiments, within Table S6, (COMBO1.7 - COMBO2.8), the optimization of the weights was further guided by PMOO (COMBO2.9.1 - COMBO2.9.3), which dynamically adjusted the contribution of each loss term during training, every 10 epochs starting on epoch 5. This ensured that no single loss dominated the optimization process, leading to a more balanced convergence. The initial configuration [Dice: 0.5, SRec: 0.33, CE: 0.17] was selected as a balanced setting to help PMOO improve performance without risking instability.

Table S6. Weight Optimization Experiments

Name	Patch Size	Batch Size	Dice Weight	SRL Weight	CE Weight	Average Epoch Time (s)	Mean Validation Dice
COMBO1.7	[96,192,192]	4	0.33	0.33	0.33	202.07	0.533
COMBO2.1	[96,192,192]	4	0.25	0.50	0.25	201.37	0.442
COMBO2.2	[96,192,192]	4	0.50	0.25	0.25	201.07	**0.551**
COMBO2.3	[96,192,192]	4	0.40	0.40	0.20	201.57	0.499
COMBO2.4	[96,192,192]	4	0.20	0.60	0.20	202.77	0.200
COMBO2.5	[96,192,192]	4	0.60	0.20	0.20	202.03	0.543
COMBO2.6	[96,192,192]	4	0.43	0.43	0.14	202.40	0.351
COMBO2.7	[96,192,192]	4	0.50	0.33	0.17	204.03	0.506
COMBO2.8	[96,192,192]	4	0.33	0.50	0.17	200.70	0.535
COMBO2.9.1	[96,192,192]	4	0.50	0.33	0.17	197.69	0.528
COMBO2.9.2	[96,192,192]	4	0.50	0.33	0.17	197.21	**0.560**
COMBO2.9.3	[96,192,192]	4	0.33	0.33	0.33	193.83	0.520

Finally, the best model was selected based on the highest Exponential Moving Average (EMA) pseudo-Dice observed on the validation set, with model checkpointing used to save this version. In addition, for computational efficiency, early stopping with a patience of 20 epochs was implemented.

In summary, as shown in Table S7, the fine-tuning process led to the selection of the following parameters for the nnInteractive model:

Table S7. Initial Weights and Training Parameters

Patch Size	Batch Size	Dice Weight	SRL Weight	CE Weight	Early Stop Patience	Total # Epochs	MVD
[96,192,192]	4	3	2	1	20	72	**0.560**

4.5 Fine-Tuning Results

4.5.1 nnInteractive Pre and Post-Fine-Tuning Results

Tables S8 and S9 present the detailed patient-level results for the nnInteractive model before and after fine-tuning, respectively. The metrics reported include Dice Similarity Coefficient (DSC), Intersection over Union (IoU), Average Surface Distance (ASD), Hausdorff Distance (HD), and Inference Time per Case (ITpC).

4.5.2 Comparative Analysis

Figure S13 presents grouped bar plots comparing the average overlap- and boundary-based metrics before and after fine-tuning, translating the patient-specific results shown in TablesS8 and S9. Due to the small sample size (9 patients), no formal statistical tests were performed, as this cohort is insufficient to draw reliable conclusions. Figure S14 illustrates segmentation results for patient AVA046, showcasing the improvements achieved through fine-tuning.

Table S8. Patient-level DSC, IoU, ASD, HD, and ITpC values for nnInteractive Pre-Fine-Tuning

Case	DSC	IoU	ASD (mm)	HD (mm)	ItpC (s)
afd	0.196	0.109	17.82	69.64	98.50
AVA046	0.076	0.040	11.10	62.40	95.54
AVA048	0.453	0.293	3.79	42.94	116.20
~~AVA070~~	~~0.183~~	~~0.101~~	~~47.10~~	~~126.73~~	~~29.50~~
AVA106	0.080	0.042	10.35	55.45	82.75
AVA116	0.110	0.058	7.93	46.82	107.18
AVA123	0.039	0.020	9.92	42.71	133.57
AVA136	0.328	0.196	5.27	40.87	11.96
AVA141	0.133	0.071	14.26	49.64	65.56
AVA146	0.153	0.083	9.65	48.94	110.97
**Mean**	0.174 ± 0.127	0.101 ± 0.084	10.01 ± 4.03	51.04 ± 9.17	91.36 ± 33.58
**Median**	0.134	0.071	10.14	49.29	98.5
**Range**	[0.039, 0.453]	[0.020, 0.293]	[3.79, 17.82]	[40.87, 69.64]	[11.96, 133.57]

Table S9. Patient-level DSC, IoU, ASD, HD, and ITpC values for nnInteractive Post-Fine-Tuning

Case	DSC	IoU	ASD (mm)	HD (mm)	ItpC (s)
afd	0.298	0.175	7.98	66.09	34.59
AVA046	0.352	0.214	6.99	68.38	33.34
AVA048	0.370	0.227	5.85	51.60	42.65
~~AVA070~~	~~0.000~~	~~0.000~~	~~Inf~~	~~Inf~~	~~10.55~~
AVA106	0.328	0.196	6.04	43.78	28.16
AVA116	0.295	0.173	4.53	45.21	36.38
AVA123	0.095	0.050	17.74	72.68	47.24
AVA136	0.185	0.102	28.37	99.55	28.30
AVA141	0.313	0.186	16.02	90.39	23.17
AVA146	0.149	0.081	18.97	51.54	40.29
**Mean**	0.265 ± 0.092	0.156 ± 0.059	11.25 ± 8.21	58.92 ± 26.40	34.90 ± 7.25
**Median**	0.298	0.175	7.49	58.84	34.59
**Range**	[0.095, 0.370]	[0.05, 0.227]	[4.53, 28.37]	[43.78, 99.55]	[23.17, 47.24]

5 QUANTITATIVE ANATOMICAL METRICS

5.1 Intramuscular Path Length

Table S10 summarizes the absolute errors for curved intramuscular paths, highlighting the discrepancies between ground truth and predicted path lengths, while Table S11 provides a similar analysis but for straight intramuscular paths, showing improved accuracy and reduced error metrics.

Figure S15 illustrates challenges in centerline extraction, particularly in bifurcated vessels.

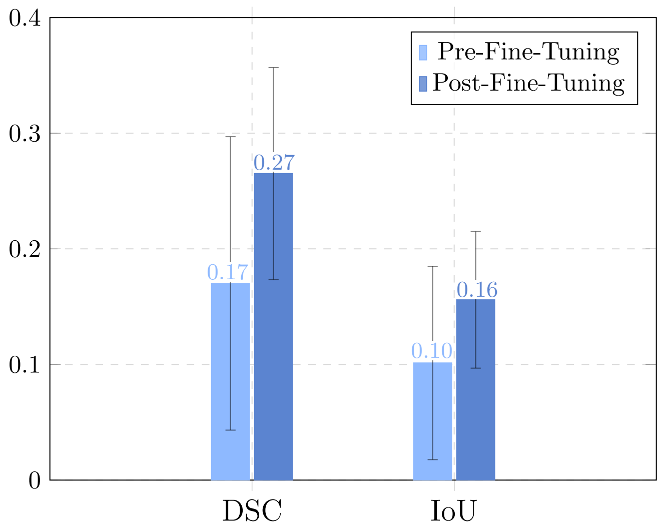

Figure S13a.

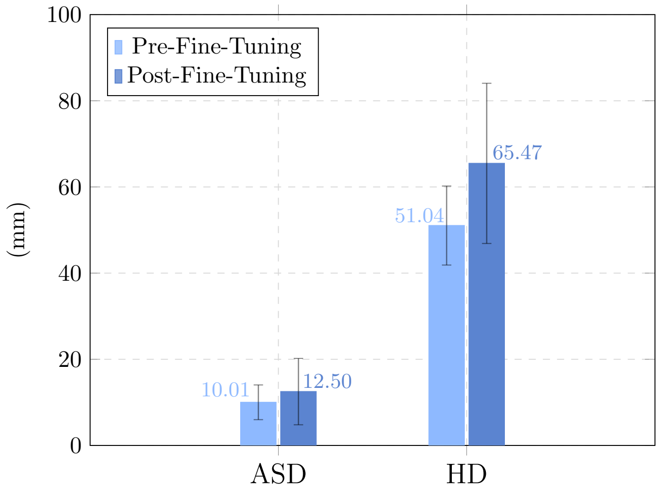

Figure S13b.

Figure S13. **a.** Comparison of averaged overlap-based metrics (DSC and IoU) and **b.** Comparison of averaged boundary-based metrics (ASD and HD) across the baseline and fine-tuned models, with mean and standard deviation indicated.

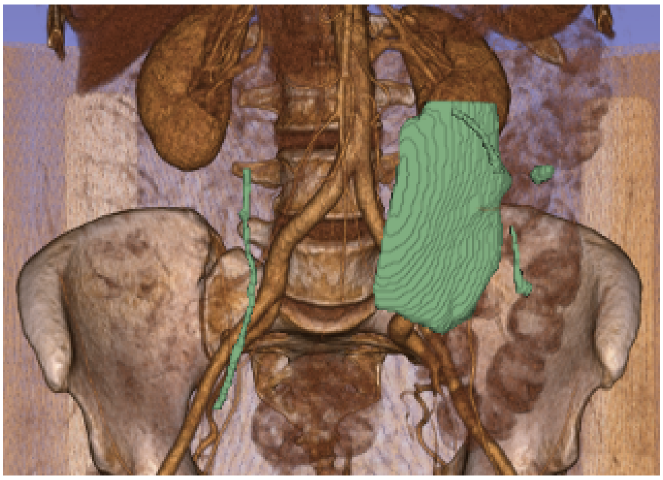

Figure S14a.

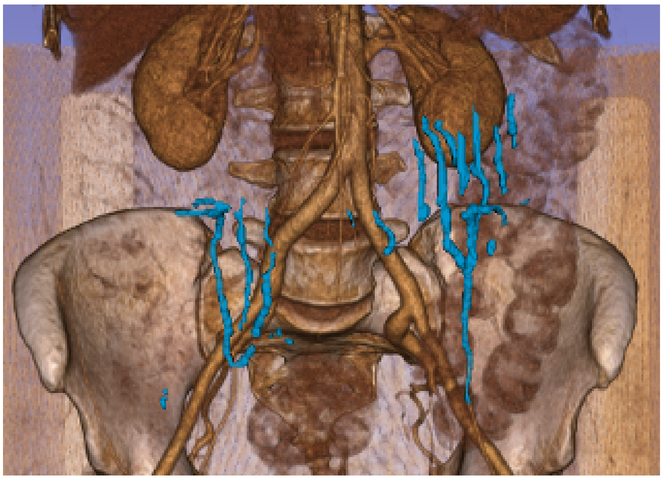

Figure S14b.

Figure S14. Example of perforator segmentation results **a.** Pre-Fine-Tuning and **b.** Post-Fine-Tuning for patient AVA046

5.2 Distance to Umbilicus

Tables S12 and S13 present the absolute errors for horizontal and vertical landmark coordinates, respectively. Summary metrics, including MdAE, MAE with IQR, and RMSE, are provided for each orientation to facilitate comparison across cases.

Table S10. Intramuscular path length analysis

Case	Ground Truth (mm)	Predicted (mm)	AE (mm)	RAE (%)
afd_1	44.89	57.93	13.04	29.05
afd_2	37.96	20.16	17.80	46.89
AVA046_1	61.34	61.74	0.40	0.65
AVA046_2	19.08	14.89	4.19	21.96
AVA046_3	12.73	15.51	2.78	21.84
AVA106_1	40.44	59.41	18.97	46.91
AVA106_2	15.25	28.75	13.50	88.52
AVA106_3	15.39	29.76	14.37	93.37
AVA116_1	56.68	44.58	12.10	21.35
AVA116_2	41.41	53.49	12.08	29.17
AVA116_3	42.06	49.56	7.50	17.83
AVA123_1	63.21	26.58	36.63	57.95
AVA141_1	14.15	19.60	5.45	38.52
AVA141_2	12.39	15.80	3.41	27.52
**MdAE (mm)**		12.09		29.11%
**MAE (IQR) (mm)**		11.59 (4.19 – 14.37)		38.68 (21.84 – 46.91) %
**RMSE (mm)**		28.38		

Table S11. Straight intramuscular path length analysis

Case	Ground Truth (mm)	Predicted (mm)	AE (mm)
afd_1	37.82	40.64	2.82
afd_2	36.80	18.60	18.20
AVA046_1	56.02	56.01	0.01
AVA046_2	16.64	14.01	2.63
AVA046_3	11.27	14.24	2.97
AVA106_1	37.71	54.67	16.96
AVA106_2	13.90	23.30	9.40
AVA106_3	14.51	27.91	13.40
AVA116_1	36.95	27.45	9.50
AVA116_2	36.19	41.36	5.17
AVA116_3	36.92	38.52	1.60
AVA123_1	57.80	25.62	31.22
AVA141_1	13.17	18.14	4.97
AVA141_2	11.98	14.91	2.93
**MdAE (mm)**		5.07	
**MAE (IQR) (mm)**		8.70 (2.82 – 13.40)	
**RMSE (mm)**		12.24	

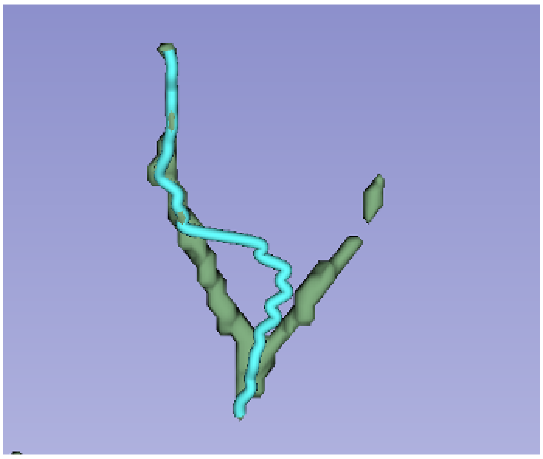

Figure S15. Illustration of centerline extraction difficulties when in a bifurcated vessel.

Table S12. Horizontal Centerline landmark localization analysis

Case	Label (GT p)	Ground Truth (mm)	Predicted (mm)	AE (mm)
afd	2 (p4)	15	28.7	13.7
	3 (p6)	34	37.4	3.4
	8 (p3)	-21	-31.5	10.5
AVA046	1 (p4)	25	36.4	11.4
	2 (p2)	-51	-50.8	0.2
	4 (p3)	-22	-32.9	10.9
AVA106	2 (p7)	84	49.2	34.8
	11 (p5)	17	15.4	1.6
AVA116	2 (p3)	-58	-33.2	24.8
	3 (p2)	-11	-19.2	8.2
	5 (p6)	61	52.6	8.4
AVA141	1 (p2)	-14	-33.7	19.7
	2 (p5)	38.5	24.7	13.8
	3 (p3)	-8	-9.2	1.2
AVA146	3 (p10)	10	26.8	16.8
	8 (p5)	-42	-21.2	20.8
**MdAE (mm)**			11.15	
**MAE (IQR) (mm)**			12.51 (7 – 17.5)	
**RMSE (mm)**			15.45	

Table S13. Vertical Centerline landmark localization analysis

Case	Label (GT p)	Ground Truth (mm)	Predicted (mm)	AE (mm)
afd	2 (p4)	-4	-3	1
	3 (p6)	-27	-35	8
	8 (p3)	-17	-22	5
AVA046	1 (p4)	22	19	3
	2 (p2)	-13	-18	5
	4 (p3)	-57	-45	12
AVA106	2 (p7)	0	-7	7
	11 (p5)	5	2	3
AVA116	2 (p3)	-13	-12	1
	3 (p2)	17	7	10
	5 (p6)	0	-19	19
AVA141	1 (p2)	-17	-16	1
	2 (p5)	-27.7	-26	1.7
	3 (p3)	-40	-41	1
AVA146	3 (p10)	-49	-48	1
	8 (p5)	-69	-51	18
**MdAE (mm)**			4.00	
**MAE (IQR) (mm)**			6.04 (1 – 8.5)	
**RMSE (mm)**			8.36	
